# Molecular basis of immune evasion by the delta and kappa SARS-CoV-2 variants

**DOI:** 10.1126/science.abl8506

**Published:** 2021-11-09

**Authors:** Matthew McCallum, Alexandra C. Walls, Kaitlin R. Sprouse, John E. Bowen, Laura E. Rosen, Ha V. Dang, Anna De Marco, Nicholas Franko, Sasha W Tilles, Jennifer Logue, Marcos C. Miranda, Margaret Ahlrichs, Lauren Carter, Gyorgy Snell, Matteo Samuele Pizzuto, Helen Y. Chu, Wesley C. Van Voorhis, Davide Corti, David Veesler

**Affiliations:** 1 Department of Biochemistry, University of Washington, Seattle, WA 98195, USA; 2 Vir Biotechnology, San Francisco, CA 94158, USA.; 3 Humabs Biomed SA, a subsidiary of Vir Biotechnology, 6500 Bellinzona, Switzerland.; 4 Division of Allergy and Infectious Diseases, University of Washington, Seattle, WA 98195, USA.; 5 Center for Emerging and Re-emerging Infectious Diseases, Division of Allergy and Infectious Diseases, Department of Medicine, University of Washington School of Medicine, Seattle, WA 98195, USA; 6 Institute for Protein Design, University of Washington, Seattle, WA 98195, USA; 7 Howard Hughes Medical Institute, Seattle, WA 98195, USA

## Abstract

SARS-CoV-2 transmission leads to the emergence of variants, including the B.1.617.2 (delta) variant of concern which is causing a new wave of infections and has become globally dominant. We show that these variants dampen the in vitro potency of vaccine-elicited serum neutralizing antibodies and provide a structural framework for describing their immune evasion. Mutations in the B.1.617.1 (kappa) and B.1.617.2 (delta) spike glycoproteins abrogate recognition by several monoclonal antibodies via alteration of key antigenic sites, including remodeling of the B.1.617.2 (delta) N-terminal domain. The ACE2 binding affinities of the B.1.617.1 (kappa) and B.1.617.2 (delta) receptor-binding domains are comparable to the Wuhan-Hu-1 isolate whereas B.1.617.2+ (delta+) exhibits markedly reduced affinity.

The ongoing spread of SARS-CoV-2, the causative agent of the COVID-19 pandemic, results in the continued emergence of variants. The B.1.351 (beta, β) variant of concern was originally described in South Africa and remains the isolate associated with the greatest magnitude of immune evasion, as measured by reduced neutralizing antibody (Ab) titers in vitro (1–3). Conversely, the B.1.1.7 (alpha, α) variant of concern, which was first detected in the United Kingdom, has a modest impact on neutralizing Ab titers but a marked enhancement in ACE2 receptor binding affinity and transmissibility, relative to the Wuhan-Hu-1 isolate, which led to worldwide dominance in the early months of 2021 (2, 4).

The SARS-CoV-2 spike (S) glycoprotein is exposed at the surface of the virus and mediates entry into host cells. S is the main target of neutralizing antibodies and the focus of most vaccines (5, 6). The S glycoprotein is subdivided into two functional subunits, designated S_1_ and S_2_, that interact non-covalently after proteolytic cleavage by furin during synthesis (5, 7, 8). The S_1_ subunit contains the receptor-binding domain (RBD), which engages the receptor ACE2 (5, 7, 9, 10), and the N-terminal domain (NTD) that recognizes attachment factors (11–13). The S_2_ subunit contains the fusion machinery and undergoes large-scale conformational changes to drive fusion of the virus and host membranes (14), enabling genome delivery and initiation of infection. Abs that bind to specific sites on the RBD (15–22), the NTD (23–26), or the fusion machinery stem helix (27–31) interfere with receptor attachment or membrane fusion. Serum neutralizing Ab titers are a correlate of protection against SARS-CoV-2 in non-human primates (32–35).

In late 2020, B.1.617 variants including B.1.617.1 (kappa, κ) and B.1.617.2 (delta, δ) were first detected in India and caused devastating epidemics before spreading globally (36, 37). The B.1.617.1 variant S harbors T95I, G142D, E154K, L452R, E484Q, D614G, P681R and Q1071H substitutions whereas the B.1.617.2 variant S carries T19R, G142D, E156G, L452R, T478K, D614G, P681R and D950N substitutions and a deletion of residues 157 and 158 (157–158del) (Table S1). Most of these mutations localize to the RBD (residues 328–531) and NTD (residues 14–305) which are the major targets of neutralizing Abs in convalescent and vaccinated individuals (18, 38), raising concerns about the efficacy of available vaccines and therapeutic monoclonal Abs (mAbs) against these variants. Moreover, the K417N mutation was detected in the B.1.617.2 lineage, known as the B.1.617.2+ (delta plus, δ+) variant, which is shared with the B.1.351 (beta, β) variant of concern and was previously shown to reduce neutralization potency of some monoclonal Abs (2, 39).

To evaluate the effect on neutralization of the mutations in the B.1.617.1, B.1.617.2, and B.1.617.2+ S glycoproteins, we compared vaccine-elicited serum neutralizing activity against ancestral G614 S and these three variant S pseudoviruses. We employed a vesicular stomatitis virus (VSV) pseudotyping system (40) with ACE2-expressing HEK293T as target cells (41). We obtained plasma samples from 37 individuals, specifically from 13, 15, and 12 individuals who received two doses of Pfizer/BioNtech BNT162b2, two doses of Moderna mRNA-1273, or a single dose of Janssen Ad26.COV2.S (Table S2). The median time after final vaccination was 11 days for Pfizer/BioNtech BNT162b2, 20 days for Moderna mRNA-1273, and 102 days for Janssen Ad26.COV2.S. All three vaccines comprise D614 in the S glycoprotein. 12 sera samples from individuals vaccinated with Janssen Ad26.COV2.S were evaluated; only 8 of these individuals are shown due to a lack of neutralization when assessed against G614 S pseudovirus for the remaining 4 samples.

Geometric mean neutralizing Ab titers (GMTs) for the Pfizer/BioNtech BNT162b2-elicited plasma were respectively reduced 3.0-, 2.4-, and 4.1-fold for B.1.617.1, B.1.617.2, and B.1.617.2+ S (GMTs 86, 110, and 63), compared to G614 S (GMT: 260) (Fig. 1A, Fig S1-S2). Moderna mRNA-1273-elicited plasma GMTs were reduced 4.1-, 2.6-, and 9.5-fold for B.1.617.1, B.1.617.2, and B.1.617.2+ S (GMTs 170, 270, and 74), respectively, compared to G614 S (GMT: 700) (Fig. 1B, Fig S1-S2). The average neutralization potency of the Janssen Ad26.COV2.S-elicited plasma was reduced 3.0-, 2.4-, and 3.5-fold for B.1.617.1, B.1.617.2, and B.1.617.2+ S (GMTs 10, 13, and 8.8), respectively, compared to G614 S (GMT: 31) (Fig. 1C, Fig S1-S2). These data demonstrate that all three variants lead to reductions in neutralization potency from vaccine-elicited Abs with B.1.617.2+ causing the greatest decrease, on par with what was observed for the B.1.351 (beta) variant of concern (1, 3, 42). Furthermore, polyclonal Abs from half of the Janssen-vaccinated individuals evaluated completely lost their ability to neutralize one or multiple variants in our assay, likely as a result of the moderate GMTs against G614 S pseudovirus, which concur with recent in vitro and in vivo data (43, 44).

Although the B.1.617.1 and B.1.617.2+ variants have greater ability to evade vaccine-elicited Ab neutralization than B.1.617.2 – a feature that might increase opportunities for breakthrough infections (37, 45, 46) – the B.1.617.2 variant became dominant worldwide by June 2021 (Fig 1D-F). B.1.617.1 and B.1.617.2 sequences were first observed in late 2020 whereas B.1.617.2+ sequences were first detected in April 2021. As of September 30, 2021, 6,806 B.1.617.1, 1,415,761 B.1.617.2, and 3,966 B.1.617.2+ sequenced genomes were deposited in GISAID (Table S1). The high incidence of B.1.617.2 is consistent with recent studies showing that the B.1.617.2 variant has enhanced transmissibility, replication kinetics and viral loads in oropharyngeal and nose-throat swabs of infected individuals relative to the ancestral Wuhan-Hu-1 virus and other variants (47–49).

We determined electron cryomicroscopy (cryoEM) structures of the B.1.617.1 and B.1.617.2 S glycoproteins to gain insights into their reduced antibody sensitivity and provide a structural framework to understand the role of shared and distinct mutations. The B.1.617.1 S structure – harboring the HexaPro stabilizing mutations (F817P, A892P, A899P, A942P, K986P, and V987P) (50), a native furin cleavage site, and with an engineered disulfide stapling the RBD in the closed conformation (S383C/D985C) (51) – was determined in complex with S309 (an RBD-targeted neutralizing mAb (15)) and S2L20 (an NTD-targeted non-neutralizing mAb (23, 52)) at 2.4 Å resolution with all three RBDs closed (Fig 2A, Fig S3A-C, Table S3). Local classification and refinements of S309 bound to the B.1.617.1 RBD were used to account for conformational dynamics and improve local resolution of this region to 3.3 Å (Fig 2A,C and Fig S3A). The B.1.617.2 S structure – harboring the F817P, A892P, A899P, A942P, V987P, Y707C, and T883C VFLIP prefusion-stabilizing mutations (53) and a native furin cleavage site – was obtained in complex with S2M11 (an RBD-targeted neutralizing mAb (19)) and S2L20 at 2.4 Å resolution with all three RBDs closed (Fig 2B, Fig S3D-F, Table S3).

The RBD is the main target of serum neutralizing activity in convalescent and vaccinated individuals and comprises several antigenic sites recognized by neutralizing Abs with a range of neutralization potencies and breadth (15–18, 38, 54, 55). E484Q, L452R, and T478K mutations are part of antigenic site I which we previously showed to be immunodominant (16–18). The B.1.617.1 S and B.1.617.2 S structures resolve the complete RBD and provide high-resolution blueprints of the residue substitutions found in these two variants (Fig 2C-D). In both structures, the L452R side chain, which is part of antigenic site Ib (18), is oriented identically (and modeled in the same rotameric configuration) to what we previously observed in the B.1.427/B.1.429 S structure (52) (Fig 2E-F). The L452R mutation reduces neutralization mediated by some clinical mAbs, such as bamlanivimab (LY-CoV555) and regdanvimab (CT-P59), due to steric alteration of this antigenic site that is incompatible with binding (Fig 2E-F) (52). The B.1.617.1 E484Q RBD mutation is located within the receptor-binding motif, at the boundary between antigenic sites Ia/Ib (18), and could affect neutralization from mAbs recognizing both subsites. The E484Q mutation is conservative, but could alter electrostatic interactions, as it substitutes the side chain carboxylic group with an amide group through replacement of an oxygen with a nitrogen atom. Residue 484 is involved in the epitopes recognized by bamlanivimab (LY-CoV555) (20, 56) and casirivimab (REGN10933) (57, 58), and both mAbs have dampened binding or neutralization potency against E484Q-harboring mutants likely due to disruption of electrostatic interactions (56, 58). The B.1.617.2 T478K RBD mutation is also located at the boundary between antigenic sites Ia/Ib but does not affect binding to mAbs that have received an emergency use authorization in the US (56, 59, 60) (Fig 2D).

Both the B.1.351 (beta) and P.1 (gamma) variants of concern harbor the E484K mutation which is associated with a reduction of vaccine-elicited neutralizing Ab titers and mAb neutralization (39, 42, 61). The E484Q mutation has also been reported to reduce serum neutralizing Ab titers alone or in combination with the L452R mutation (42, 58, 62). The effect of the T478K mutation on polyclonal Ab responses has not been characterized as thoroughly as the L452R and E484Q substitutions. The recurrent emergence of mutants carrying residue substitutions at position 452 and 484 underscores the apparent hyperfocusing of neutralizing antibody responses at these antigenic subsites and provides a possible explanation for their acquisition in multiple SARS-CoV-2 lineages. Our structural data integrated with previous studies (15, 48, 60, 63) emphasize that S309 is a variant-proof neutralizing mAb which is thus far resilient to the emergence of SARS-CoV-2 variants.

We determined the impact of the RBD mutations present in the B.1.617.1, B.1.617.2, and B.1.617.2+ on binding affinity for the ACE2 receptor to assess potential impact on a component of viral transmissibility. The B.1.617.1 (L452R/E484Q) and B.1.617.2 (L452R/T478K) RBDs recognized immobilized ACE2 with roughly similar efficiency to the wildtype RBD, as observed by ELISA (Fig 2G, Fig S4, Table S4). ACE2 binding to the B.1.617.2+ (K417N/L452R/T478K) and B.1.1.7 (N501Y) RBDs was respectively much weaker and much tighter than for any other variants evaluated in our assay, in agreement with the enhanced affinity conferred by N501Y (4, 64) (Fig 2G, Table S4). We confirmed these results using surface plasmon resonance (Fig 2H, Fig S4, Table S4) and biolayer interferometry (Fig 2I, Fig S4, Table S4) binding analysis of the monomeric human ACE2 ectodomain to immobilized RBDs, indicating that the B.1.617.1 and B.1.617.2 RBDs interact with ACE2 with roughly comparable affinity to the wildtype RBD whereas the B.1.617.2+ RBD is severely attenuated. These data concur with evaluation of the effect of individual residue mutations on ACE2 binding using deep-mutational scanning of yeast-displayed RBDs (64), the positioning of the R452 and K478 side chains away from the ACE2-binding interface, and the conservative nature of the E484Q substitution (Fig 2J-K). Finally, these data point to the key contribution of the salt bridge formed between the K417_SARS-CoV-2_ and D30_ACE2_ side chains for receptor engagement (Fig 2J-K), in agreement with previous studies (65, 66). Since none of the B.1.617.1 and B.1.617.2 RBD mutations increase ACE2 binding markedly and all of them reside in the most immunogenic antigenic site, we suggest that they might have emerged mainly as a result of antibody-mediated selective pressure to reduce immune recognition.

Although multiple antigenic sites are present at the surface of the NTD, a single supersite of vulnerability is targeted by neutralizing Abs elicited upon infection and vaccination (23, 24, 26). This antigenic supersite (designated site i) comprises the NTD N-terminus (residues 14–20), a β-hairpin (residues 140–158), and a loop (residues 245–264). To improve the cryoEM map resolution of the B.1.617.1 and B.1.617.2 NTDs bound by S2L20 and visualize the structural changes associated with their respective constellation of mutations, we used focused 3D classification and local refinement. Two of the three B.1.617.1 NTD mutations, G142D and E154K, map to the supersite β-hairpin (Fig 3A, Table S1) and we have previously shown that G142D abrogates binding of 3 out of 5 NTD-specific neutralizing mAbs tested (23). The B.1.617.1 T95I substitution occurs outside the antigenic supersite and is unlikely to contribute to immune evasion significantly (Fig 3A). All of the B.1.617.2 mutations are found within the antigenic supersite: T19R, G142D, E156G, and 157–158del (Fig 3B). The T19R substitution abrogates the glycosylation sequon at position N17, as supported by the lack of a resolved glycan at this position in the cryoEM map. We previously showed that T19A, which also removes the N17 glycosylation sequon, decreased binding to 4 out 5 NTD neutralizing mAbs tested (23). Residues 156–158 participate in the supersite β-hairpin and their mutation/deletion in the B.1.617.2 NTD lead to striking structural remodeling: residues 151–159 adopt an alpha-helical conformation whereas this segment is mostly β-stranded in the absence of this mutation/deletion (Fig 3B and Fig S5). Based on these findings, we hypothesized that B.1.617.1 and B.1.617.2 variant S glycoproteins would escape recognition by most neutralizing NTD Abs (Fig 3C & 3D).

We therefore evaluated binding of a panel of neutralizing NTD antibodies to variant S ectodomain trimers by ELISA (Fig 4A, Fig S6). Out of 11 neutralizing mAbs tested, we observed a 10-fold or greater reduction in binding for 8, 10, 10, 10, 3, and 11 mAbs to B.1.617.1, B.1.617.2, B.1.1.7, B.1.351, P.1, and B.1.427/B.1.429 S as a result of the introduced mutations and deletions whereas the non-neutralizing S2L20 mAb efficiently recognized all S trimers through binding to antigenic site iv (23), confirming proper folding (Fig 4A). Collectively, these data indicate that NTD-specific neutralizing Abs might exert a selective pressure participating in evolution of the antigenic supersite leading to neutralization escape in numerous variants (67–69), including B.1.617.1 and B.1.617.2. The diversity of molecular solutions observed in SARS-CoV-2 variants to evade NTD-targeted neutralizing Ab responses further underscores the plasticity of this domain.

S2X303 stood out due to its greater cross-reactivity with variants, including B.1.351, P.1, B.1.617.1 and to a lesser extent B.1.617.2, compared to all other neutralizing mAbs evaluated (Fig 4A). To provide a structural framework for understanding the S2X303 binding breadth, we characterized its Fab fragment bound to B.1.617.1 S using cryoEM (Fig 4B). Focused classification and local refinement of the S2X303-bound NTD yielded a map at 3.5 Å resolution revealing the recognition mode of this mAb (Fig 4C, Fig S3G-I, Table S3).

S2X303 recognizes the NTD with an angle of approach almost orthogonal relative to several previously described NTD-specific neutralizing mAbs and its epitope only partially overlaps with the NTD antigenic supersite (23). Specifically, the S2X303 complementary determining region 3-dominated paratope exclusively contacts residues 123–125 (along with the glycan at position N122), that are part of the NTD galectin-like distal β-sheet, and residues 144–154 within the supersite β-hairpin (Fig 4D-E). As a result, S2X303 defines a new class of NTD-specific mAbs that differs from S2X333 (23), a canonical ultrapotent NTD-specific mAb, and from P008_056 (70), which interferes with biliverdin binding, with respect to their footprints and angles of approach (Fig 4E). Moreover, S2X303 exhibits better cross-reactivity compared to all other mAbs and can bind to a large number of variants although not all of the ones tested (Fig 4A). Furthermore, we found that although S2X303 neutralized G614 S pseudovirus, it could not neutralize the B.1.617.1 S and B.1.617.2 S pseudoviruses, putatively reflecting enhanced off-rate and reduced binding affinity not detected by ELISA (Fig S7).

Mutations found in the B.1.617.1 and B.1.617.2 variants mediate immune evasion by eroding infection- and vaccine-elicited serum neutralizing Ab titers due to structural alteration present in major antigenic sites within the RBD and NTD (37, 71, 72). Neither of the fusion machinery B.1.617.1 Q1071H or the B.1.617.2 D950N substitutions are part of epitopes known to be recognized by neutralizing Abs. The B.1.617.2+ variant is associated with a severe dampening of neutralizing Ab titers due to the additional K417N RBD substitution, also found in the B.1.351 variant of concern (1, 2, 39). However, the deleterious effect of this mutation on ACE2 binding and absence of the compensatory N501Y mutation found in B.1.351 (4) might be associated with a fitness cost, putatively explaining the small number of genomes detected for this variant. Based on the roughly comparable ACE2 binding affinities of the B.1.617.1, B.1.617.2 and the Wuhan-Hu-1 RBDs, we propose that other factors contribute to the enhanced transmissibility of the B.1.617.2 variant. The P681R mutation found in the B.1.617.1 and B.1.617.2 variants was recently shown to enhance cleavage at the S_1_/S_2_ boundary, cell-cell fusion and replication kinetics (48, 49, 72, 73). Since the S_1_/S_2_ cleavage site is key for transmission and pathogenicity (74, 75), it appears likely that this mutation contributes to the success of these lineages. Furthermore, the L452R mutation, which augments stability and expression (64), increases viral replication kinetics relative to the Wuhan-Hu-1 virus (76). The enhanced ability of B.1.1.7 to antagonize host innate immunity through upregulation of orf6 and orf9b was suggested to have participated in the success of this variant (77). It is possible that B.1.617.2 evolved a similar strategy to reach global domination warranting further studies to uncover the contribution of innate immune antagonism to the continued emergence of variants with greater transmissibility.

S309, the parent mAb of sotrovimab which has received an emergency use authorization from the FDA, is unaffected by antigenic drift observed in variants of concern and interest, including B.1.617.1 and B.1.617.2(+), due to recognition of a conserved RBD epitope (15, 48, 60, 63), as illustrated here. The recent discovery of multiple additional conserved antigenic sites recognized by RBD-specific mAbs with (near) pan-sarbecovirus neutralizing activity (16, 17, 21, 78–80) and the anticipated continued emergence of SARS-CoV-2 variants motivate the clinical development and deployment of some of these mAbs for prophylaxis and for treatments of unvaccinated individuals or breakthrough infections in at-risk patients. Moreover, next-generation vaccine candidates have recently been described to elicit broad sarbecovirus immunity (32, 42, 81–83), holding the promise to be resilient to the emergence of SARS-CoV-2 variants and of new zoonotic sarbecoviruses. Looking forward, the discovery of broadly neutralizing mAbs targeting the fusion machinery makes tangible the development of a universal β-coronavirus vaccine (27–30, 84).

## Supplementary Material

FigureS1

FigureS2

FigureS3

FigureS4

FigureS5

FigureS6

FigureS7

Supplementary materials all

MDAR checklist

## Figures and Tables

**Figure 1.**
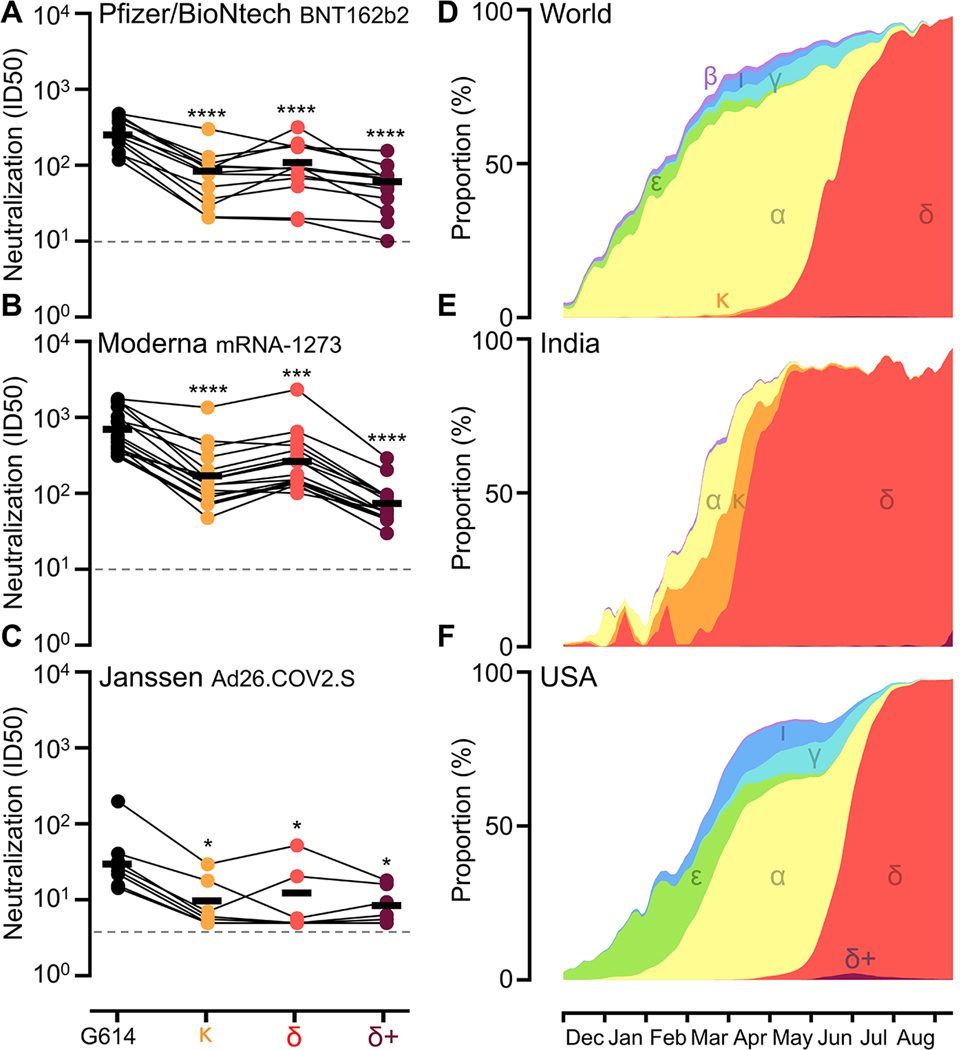
Neutralization of pseudotyped viruses harboring SARS-CoV-2 G614 S, B.1.617.1 S, B.1.617.2 S, or B.1.617.2+ S and incidence of variants. **(A-C)** Pairwise connected 50% inhibition concentrations (IC50s) for each individual against each variant for Pfizer/BioNtech BNT162b2 (A), Moderna mRNA-1273 (B), or Janssen Ad26.COV2.S (C) vaccinee sera (Fig. S1 and Table S2). Data are an average of n = 2 replicates and are representative of at least two independent assays with distinct batches of pseudoviruses. Dashed line indicates the limit of detection for the assay. Means (shown as thick black horizontal lines) were compared against G614 by two-way ANOVA (Dunnet’s test); *, p<0.05; ***, p<0.001; ****, p<0.0001. **(D-F)** Incidence (7-day average) of variants of concern and variants of interest as a proportion of viruses sequenced in the world (D), India (E), and the USA (F) deposited to GISAID (analyzed using outbreak.info) from December 1, 2020 to September 30, 2021. B.1.1.7 (alpha, α), B.1.351 (beta, β), P.1 (gamma, γ), B.1.617.2 (delta, δ) including AY.3-AY.31, B.1.526 (iota, ι), B.1.427/B.1.429 (epsilon, ε), B.1.617.1 (kappa, κ), and B.1.617.2+ (delta+, δ+) including AY.1 and AY.2 are shown in yellow, light purple, cyan, red, blue, green, orange, and dark purple, respectively.

**Figure 2.**
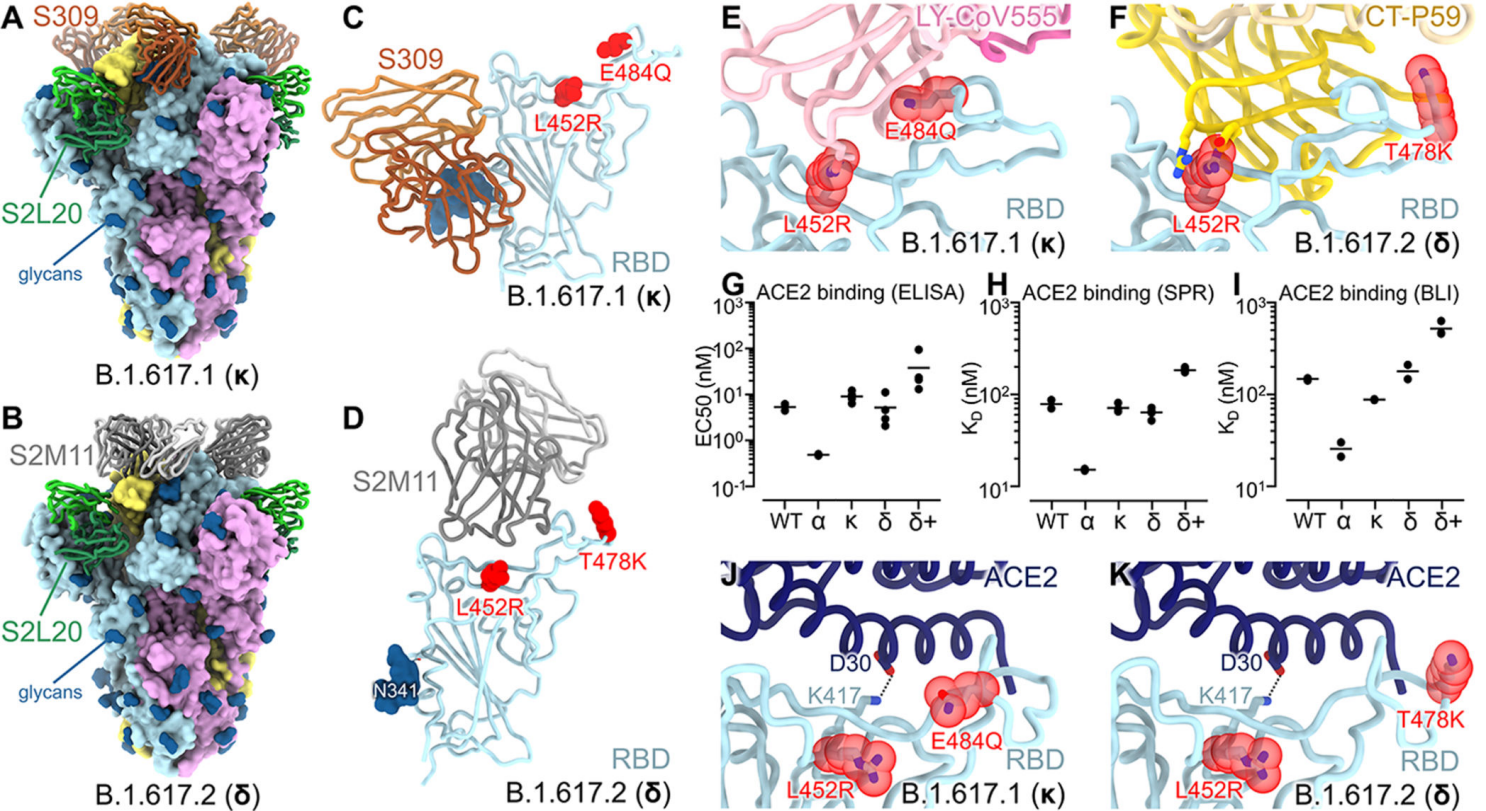
CryoEM structures of the SARS-CoV-2 B.1.617.1 and B.1.617.2 S ectodomain trimers and analysis of ACE2 binding. **(A-B)** Structure of the B.1.617.1 (A) and B.1.617.2 (B) S trimer (surface rendering) bound to the S2L20 and S309 (A) or S2M11 (B) Fabs (ribbons). SARS-CoV-2 S protomers are colored pink, cyan, and gold, whereas the S2L20 Fab heavy and light chains are colored dark and light green, respectively. The S309 Fab heavy and light chains are colored dark and light orange, respectively (A). The S2M11 Fab heavy and light chains are colored dark and light gray, respectively (B). Only the Fab variable domains are resolved and therefore modeled in the map. N-linked glycans are rendered as dark blue spheres. (**C**) Zoomed in view of the S309-bound B.1.617.1 RBD with L452R and E484Q shown as red spheres. (**D**) Zoomed in view of the S2M11-bound B.1.617.2 RBD with L452R and T478K shown as red spheres. (**E**) Superimposition of the LY-CoV555–bound SARS-CoV-2 RBD structure (PDB 7KMG) on the SARS-CoV-2 B.1.617.1 S cryoEM structure show that L452R would clash with the mAb and E484Q would disrupt electrostatic interactions. (**F**) Superimposition of the CT-P59–bound SARS-CoV-2 RBD structure (PDB 7CM4) on the SARS-CoV-2 B.1.617.2 S cryoEM structure show that L452R would sterically clash with the mAb. (**G**) Enzyme-linked immunosorbant assay (ELISA) binding analysis of the SARS-CoV-2 wildtype, B.1.1.7 (α), B.1.617.1 (κ), B.1.617.2 (δ), and B.1.617.2+ (δ+) RBDs to immobilized human ACE2 ectodomain (residues 1–615) shown as 50% effective concentrations (EC_50_). Data from two biological replicates are shown with 2–4 technical replicates each. (**H**) Surface plasmon resonance (SPR) binding affinity analysis of the human ACE2 ectodomain (residues 1–615) for immobilized biotinylated wildtype, B.1.1.7 (α), B.1.617.1 (κ), B.1.617.2 (δ), and B.1.617.2+ (δ+) RBDs. Data from two biological replicates are shown with 2–6 technical replicates each. (**I**) Biolayer interferometry (BLI) binding analysis of the human ACE2 ectodomain (residues 1–615) to immobilized biotinylated SARS-CoV-2 wildtype, B.1.1.7 (α), B.1.617.1 (κ), B.1.617.2 (δ), and B.1.617.2+ (δ+) RBDs. Data from two biological replicates are shown with 1–2 technical replicates each. (**J-K**) Superimposition of the ACE2-bound SARS-CoV-2 RBD structure (PDB 6VW1) on the SARS-CoV-2 B.1.617.1 (J) and B.1.617.2 (K) S cryoEM structures show that L452R and T478K point away from the interface with ACE2, while K417 contacts D30 from ACE2.

**Figure 3.**
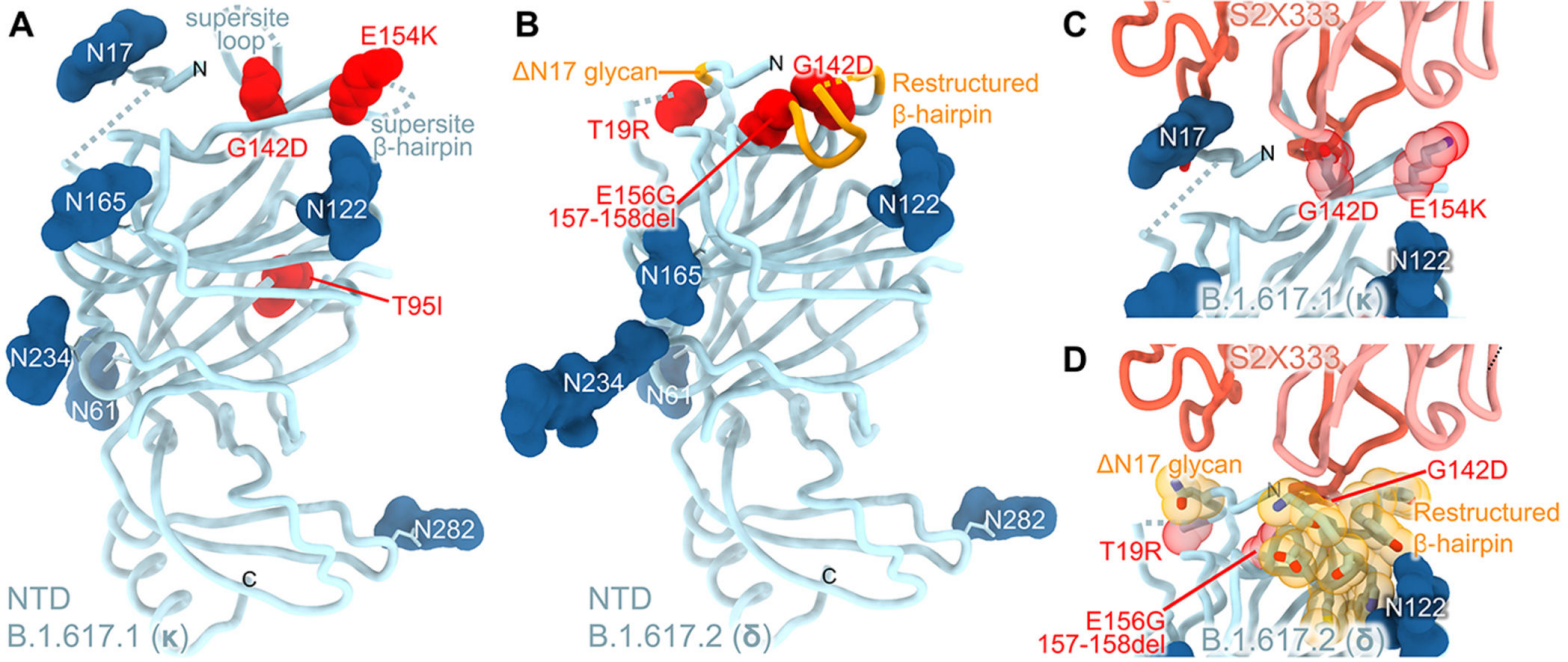
Remodeling of the NTD antigenic supersite in the B.1.617.1 and B.1.617.2 S variants. **A-B,** Ribbon diagrams of the B.1.617.1 (A) and B.1.617.2 (B) NTDs in the same orientation. Mutated residues are rendered as red spheres and N-linked glycans are shown as dark blue surfaces. Segments with notable structural changes as a consequence of these mutated residues are shown in orange and labeled. **C-D,** Zoomed-in views of the B.1.617.1 (C) and B.1.617.2 (D) NTD antigenic supersites highlighting incompatibility with recognition by the S2X333 mAb (23) (used here as an example of prototypical NTD neutralizing mAb). N- and C- termini are labeled.

**Figure 4.**
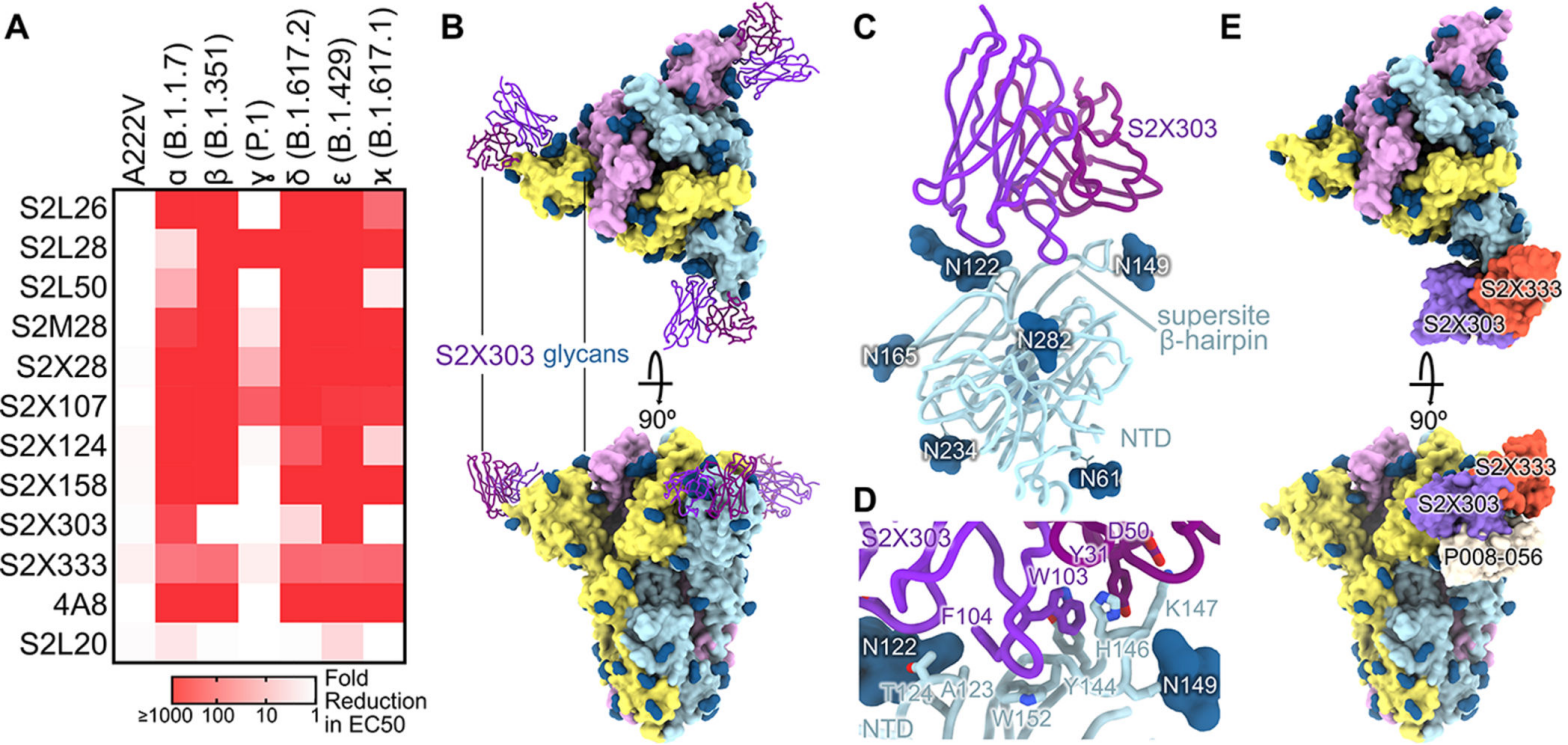
S2X303 defines a subclass of site i NTD mAbs cross-reacting with several variants. (**A**) Binding of a panel of 11 neutralizing (antigenic site i) and 1 non-neutralizing (S2L20, antigenic site iv) NTD-specific mAbs to recombinant SARS-CoV-2 S variants analyzed by ELISA displayed as a heat map (relative to wildtype Wuhan-Hu-1 binding). (**B**) Structure of the B.1.617.1 S trimer (surface rendering) bound to the S2X303 Fab fragment (ribbons) shown in two orthogonal orientations. SARS-CoV-2 S protomers are colored pink, cyan, and gold, whereas the S2X303 Fab heavy and light chains are colored dark and light purple, respectively. Only the Fab variable domains are resolved and therefore modeled in the map. N-linked glycans are rendered as dark blue spheres. (**C**) Ribbon diagram of the S2X303-bound SARS-CoV-2 B.1.617.1 NTD. (**D**) Zoomed-in view of the S2X303-bound B.1.617.1 NTD with key residues involved in the interface shown as sticks. (**E**) Structure of the S trimer bound to the S2X303 overlaid with S2X333 and P008–056 antibodies (PDB IDs 7LXW and 7NTC, respectively) shown as a surface rendering. S is colored as in (B); S2X303, S2X333, and P008–056 are shown in purple, orange, and light grey, respectively.

## Data Availability

The cryoEM maps and coordinates have been deposited to the Electron Microscopy Databank and Protein Data Bank with accession numbers listed in Table S3. Materials generated in this study will be made available on request after signing a materials transfer agreement with Vir Biotechnology or the University of Washington.
