# Supplementary figures and images for: Molecular basis of immune evasion by the delta and kappa SARS-CoV-2 variants
Source: Science. Author manuscript; Available in PMC 2025 Jul 9. (PMC12240541; doi:10.1126/science.abl8506)

Pfizer - BNT162b2

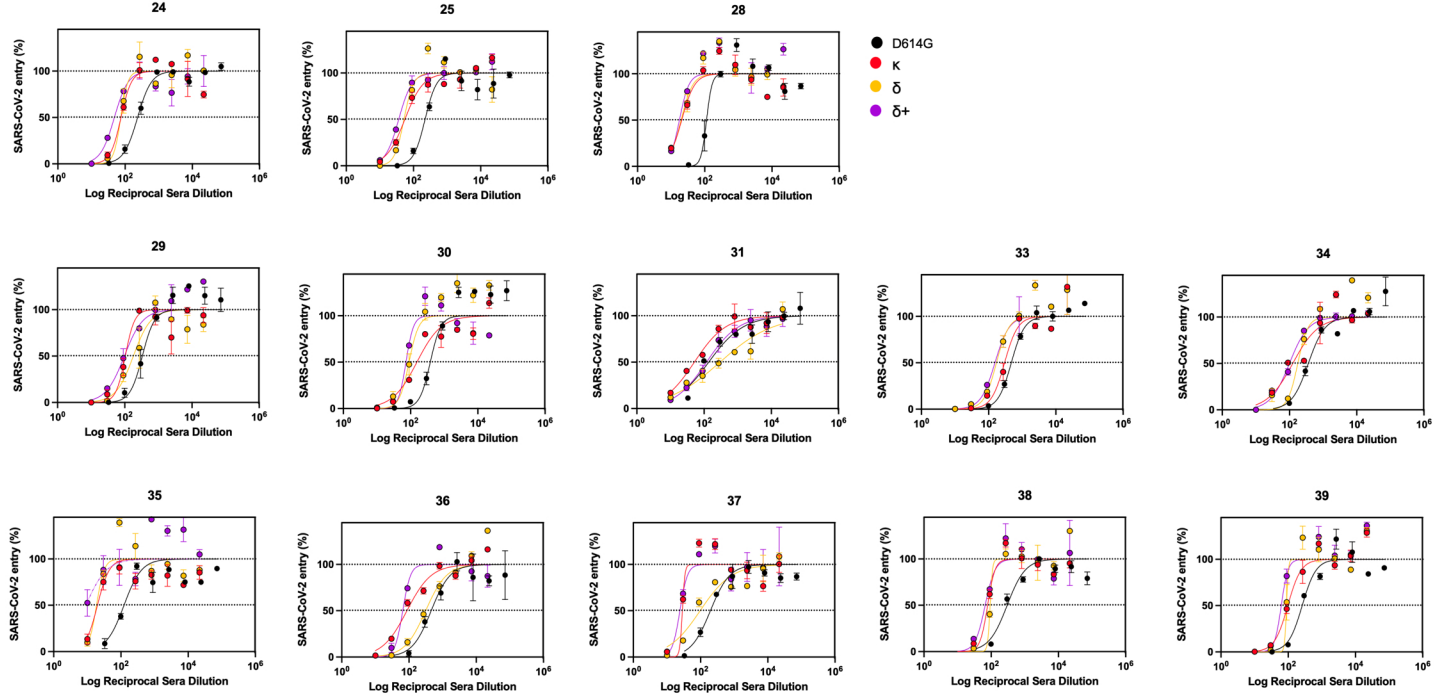

Moderna - mRNA-1273

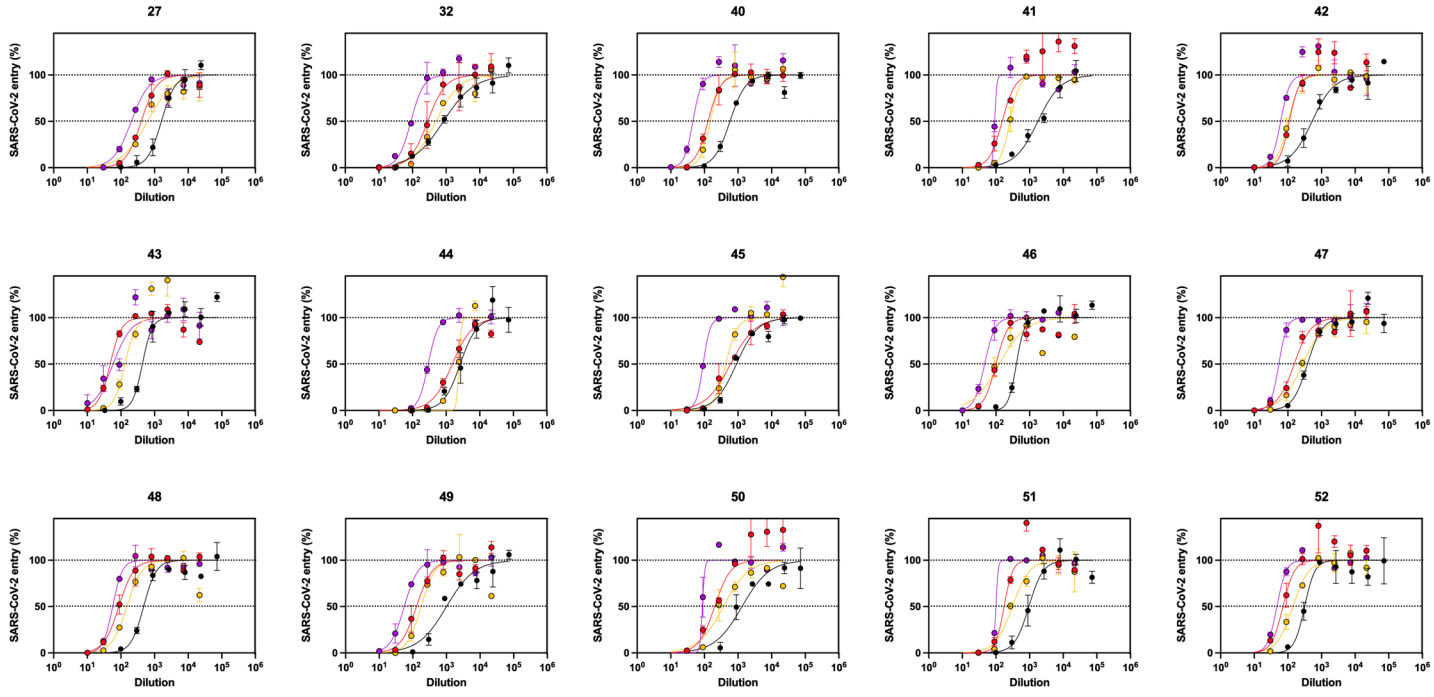

Janssen - Ad26.COVS.2S

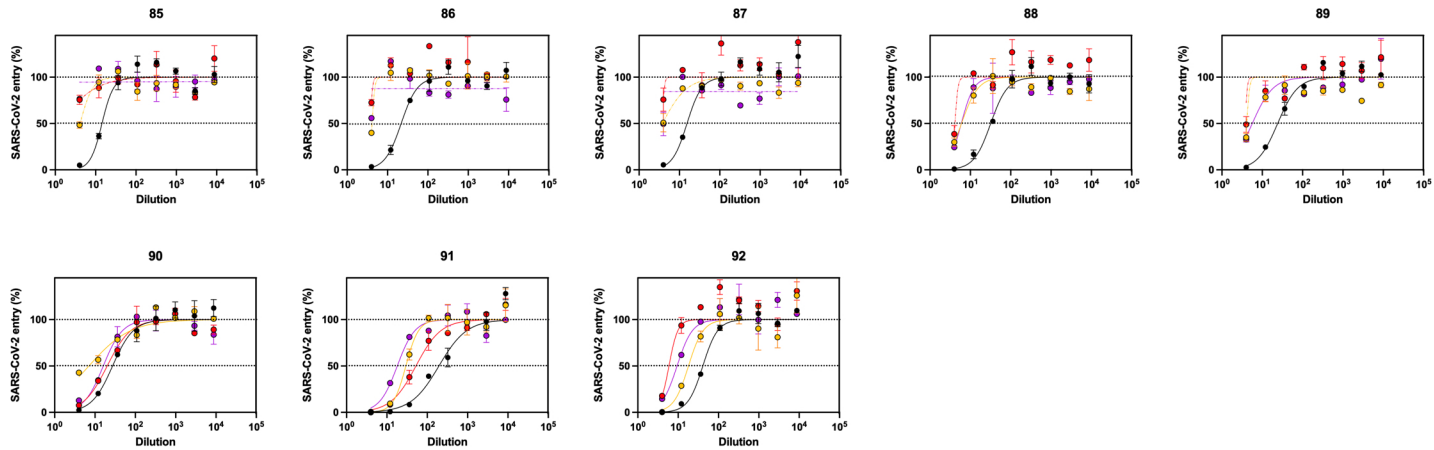

Supplement: FigureS1 [file NIHMS2082814-supplement-FigureS1.pdf]

G614

K

$\delta$

$\delta^+$

250 kDa

130 kDa

95 kDa

70 kDa

55 kDa

34 kDa

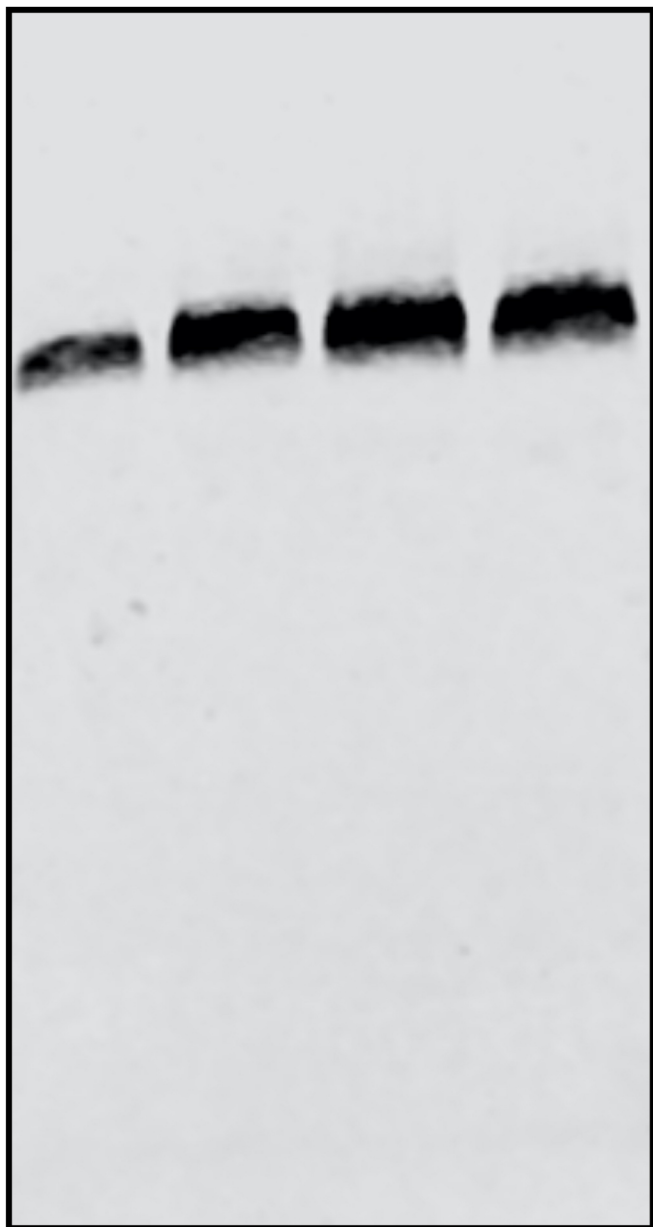

Supplement: FigureS2 [file NIHMS2082814-supplement-FigureS2.pdf]

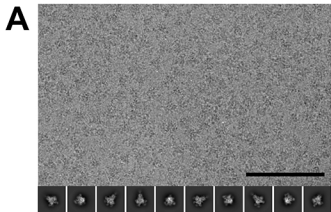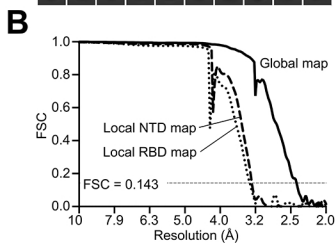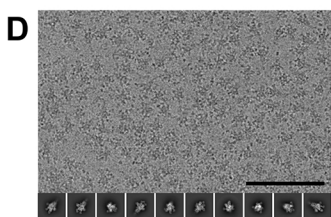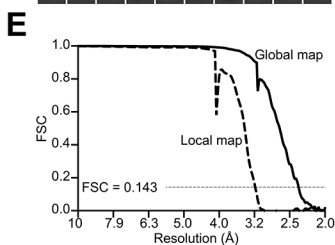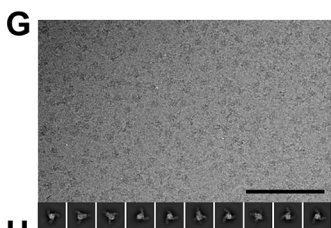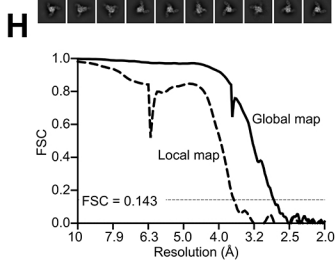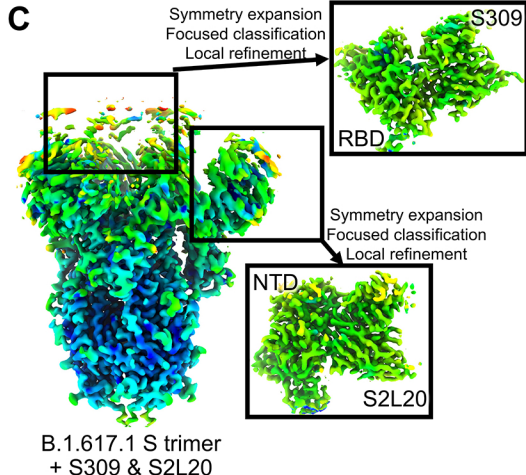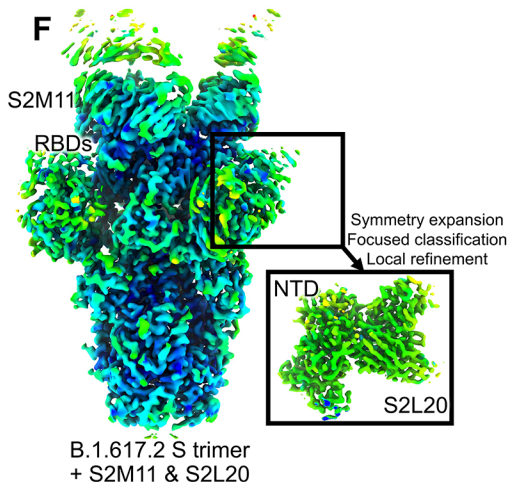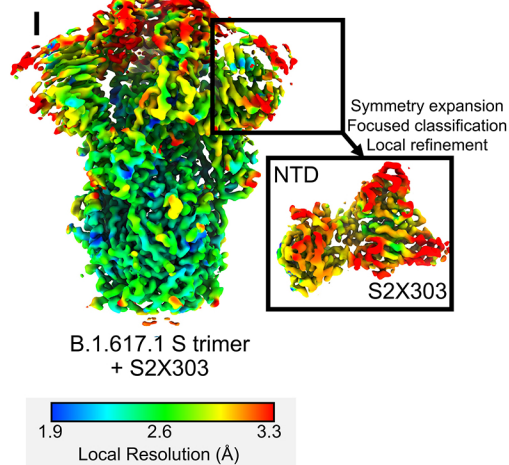

Supplement: FigureS3 [file NIHMS2082814-supplement-FigureS3.pdf]

# A, ELISA

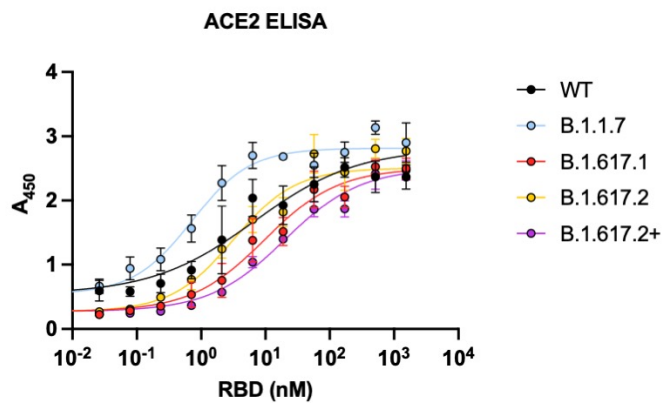

# B, SPR

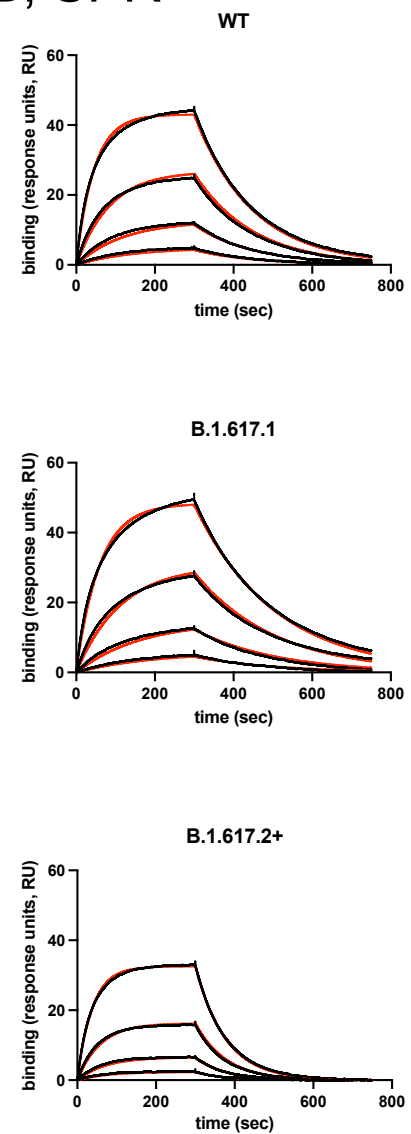

# C, BLI

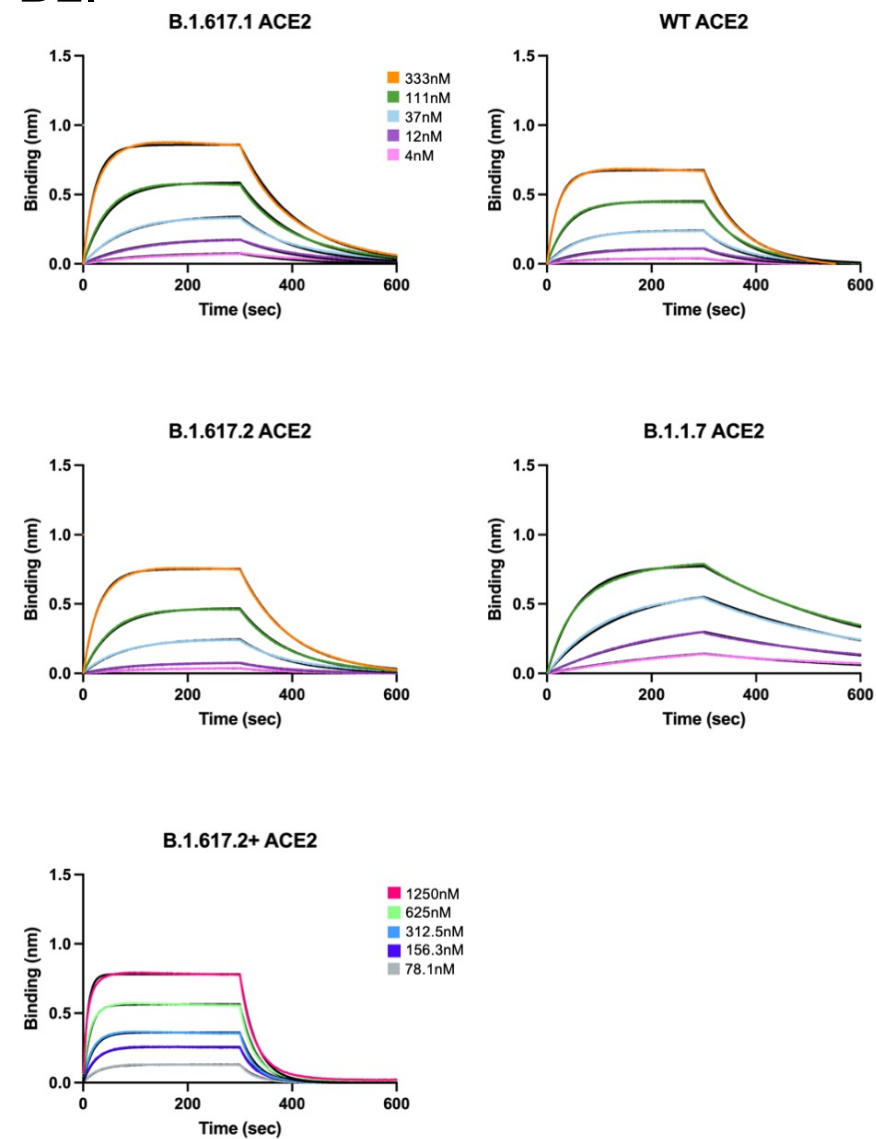

Supplement: FigureS4 [file NIHMS2082814-supplement-FigureS4.pdf]

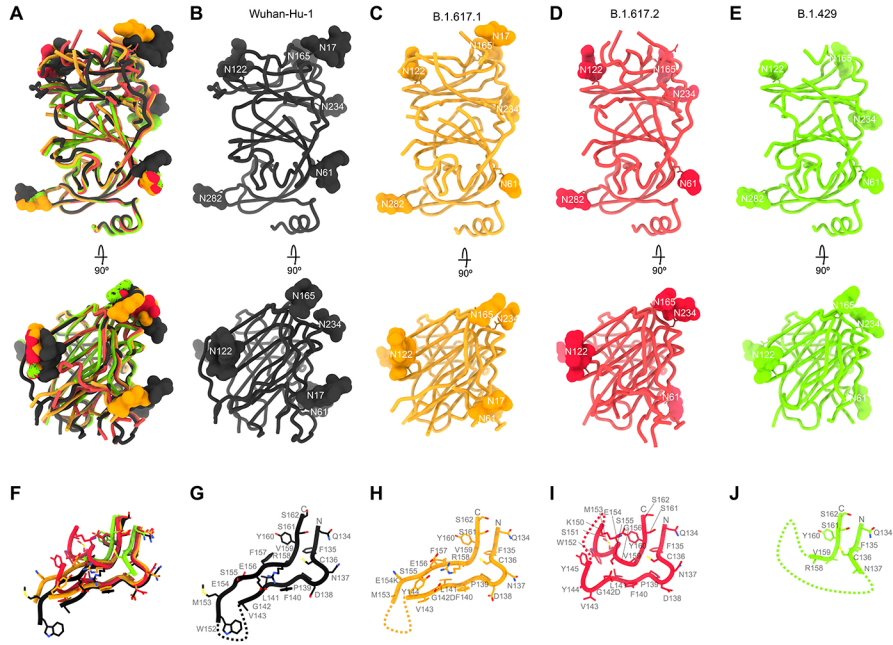

Supplement: FigureS5 [file NIHMS2082814-supplement-FigureS5.pdf]

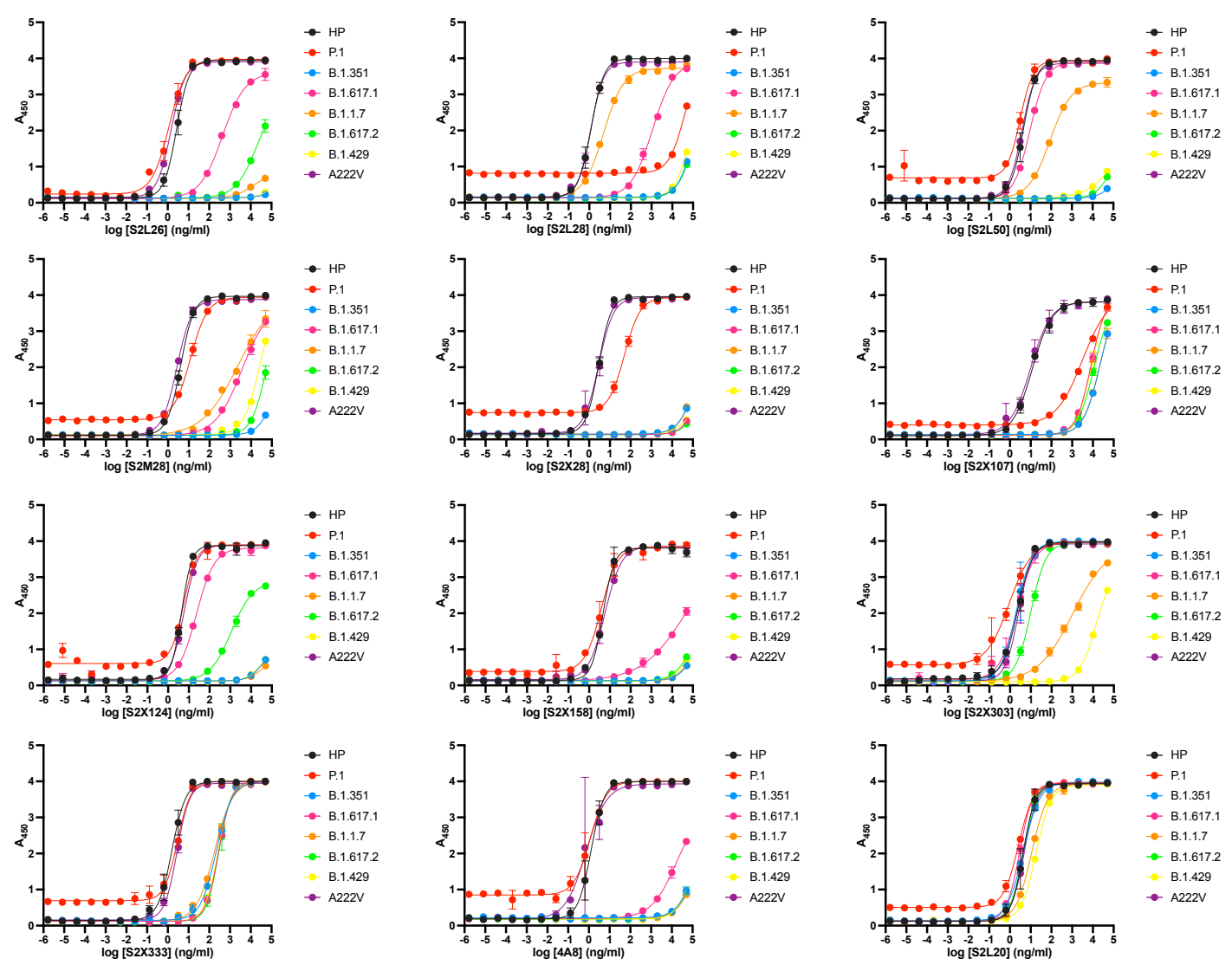

Supplement: FigureS6 [file NIHMS2082814-supplement-FigureS6.pdf]

**G614**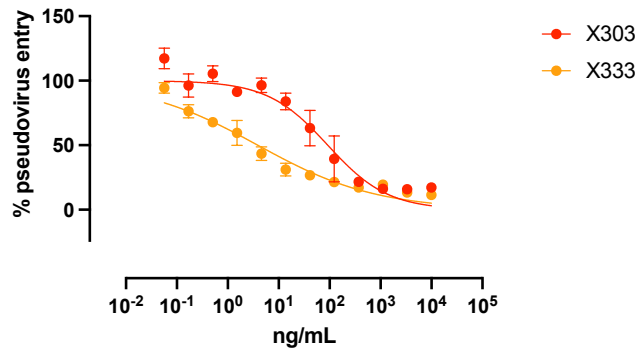**B.1.1.7**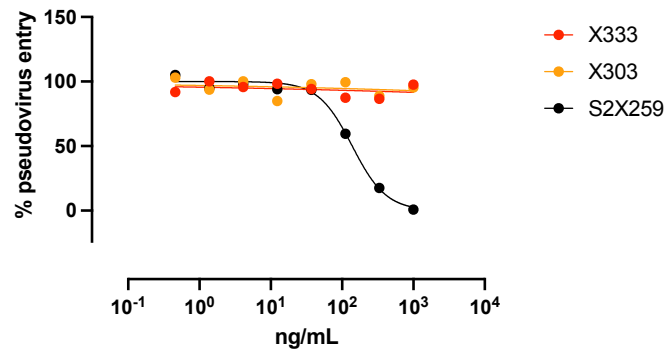**B.1.617.1**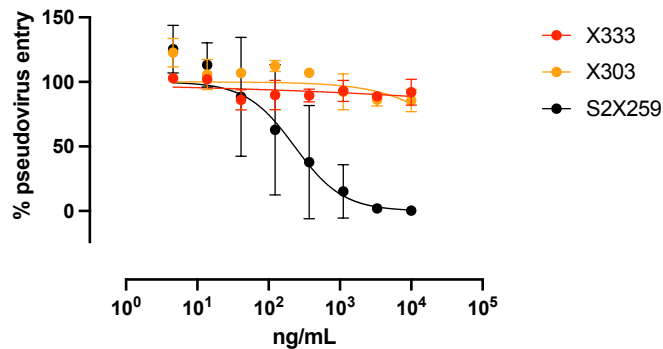**B.1.617.2**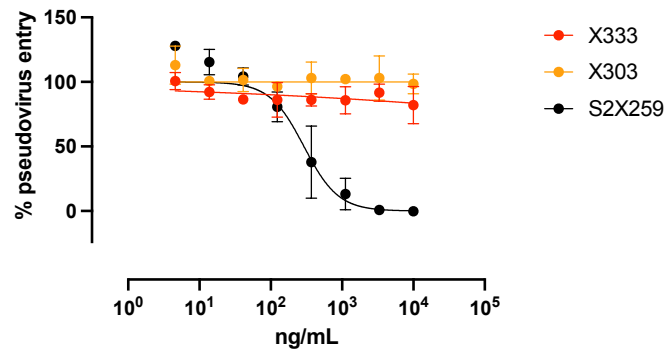

Supplement: FigureS7 [file NIHMS2082814-supplement-FigureS7.pdf]
