# Supplementary material for: Molecular basis of immune evasion by the delta and kappa SARS-CoV-2 variants
Source: Science. Author manuscript; Available in PMC 2025 Jul 9. (PMC12240541; doi:10.1126/science.abl8506)
Supplement: Supplementary materials all [file NIHMS2082814-supplement-Supplementary_materials_all.pdf]

Science
AAAS

Supplementary Materials for

Molecular basis of immune evasion by the Delta and Kappa SARS-CoV-2 variants

Matthew McCallum *et al.*

Corresponding author: David Veesler, dveesler@uw.edu

Science **374**, 1621 (2021)
DOI: 10.1126/science.abl8506

The PDF file includes:

Materials and Methods
Figs. S1 to S7
Tables S1 to S4
References

Other Supplementary Material for this manuscript includes the following:

MDAR Reproducibility Checklist

Variant incidence analysis

Average daily prevalence for B.1.1.7, B.1.351, P.1, B.1.617.2 (including AY.3-AY.31), B.1.526, B.1.427/B.1.429, and B.1.617.2+ (including AY.1 and AY.2) were obtained from GISAID (using outbreak.info) and plotted using GraphPad PRISM software (version 9.2.0).

Sample donors

Blood samples were collected from participants who had received both doses of the Pfizer's BNT162b2 vaccine or Moderna's mRNA-1273 vaccine and were 7–30 days post second dose. Individuals were enrolled in the UWARN: COVID-19 in WA study at the University of Washington in Seattle, WA. This study was approved by the University of Washington Human Subjects Division Institutional Review Board (STUDY00010350). The J&J/Janssen Ad26.COV2.S vaccine samples were collected as part of the HAARVI study and was approved by the University of Washington Human Subjects Division Institutional Review Board (STUDY00000959). Baseline sociodemographic and clinical data for these individuals are summarized in Table S2.

Recombinant expression of mAbs

Recombinant mAbs were expressed in ExpiCHO cells at 37 °C and 8% CO2. Cells were transfected using ExpiFectamine. Transfected cells were supplemented 1 day after transfection with ExpiCHO Feed and ExpiFectamine CHO Enhancer. Cell culture supernatant was collected eight days after transfection and filtered through a 0.2 μm filter. Recombinant antibodies were affinity purified on an ÄKTA xpress FPLC device using 5 mL HiTrapTM MabSelectTM PrismA columns followed by buffer exchange to Histidine buffer (20 mM Histidine, 8% sucrose, pH 6) using HiPrep 26/10 desalting columns.

Variant construct generation

The WT, B.1.1.7, B.1.617.1, B.1.617.2, and B.1.617.2+ SARS-CoV-2 RBD construct were synthesized by GenScript into pCMVR with an N-terminal mu-phosphatase signal peptide, and a C-terminal octa-histidine tag (GHHHHHHHH) and an avi tag (GLNDIFEAQKIEWHE)). The boundaries of the construct are N-328-RFPN-331 and 528-KKST-531.

The hACE2 construct was previously synthesized by Twist into pTwist+CMV(residues 1-615 with a C-terminal avi tag-10xHis-GGG-tag, and N-terminal signal peptide) (*64*).

The SARS-CoV-2 S ectodomain with hexapro mutations (F817P, A892P, A899P, A942P, K986P, and V987P) (*50*), S383C/D985C mutations (*51*), the native furin cleavage site (RRAR), and B.1.617.1 spike mutations (T95I, G142D, E154K, L452R, E484Q, D614G, P681R and Q1071H) was synthesised by GenScript into pCMV with a C-terminal foldon and avi tag followed

by an octa-histidine tag. The SARS-CoV-2 S ectodomain with the F817P, A892P, A899P, A942P, V987P, Y707C, and T883C VFLIP mutations (*53*), the native furin cleavage site (RRAR), and B.1.617.2 spike mutations (T19R, G142D, E156G, T478K, and D950N substitutions and a deletion of residues 157 and 158) was synthesised by GenScript into pCMV with a C-terminal avi tag followed by an octa-histidine tag. The SARS-CoV-2 S ectodomain with hexapro mutations and P.1 spike mutations (L18F, T20N, P26S, D138Y, R190S, K417T, E484K, N501Y, D614G, H655Y, T1027I, and V1176F) was synthesised by GenScript into pCMVR with foldon, a C-terminal avi tag followed by an octa-histidine tag. The SARS-CoV-2 S ectodomain with the 2P mutations and B.1.1.7 spike mutations (del69/70, del144/145, N501Y, A570D, D614G, P681H, T716I, S982A, and D1118H) was synthesised by GenScript into pCMV with a C-terminal foldon and avi tag followed by an octa-histidine tag. The SARS-CoV-2 S ectodomain with the 2P mutations and B.1.351 spike mutations (D80A, D215G, 242-244del, R246I, K417N, E484K, N501Y, D614G, and A701V) was synthesised by GenScript into pCMV with a C-terminal foldon and avi tag followed by an octa-histidine tag.

Production of recombinant glycoproteins

hACE2 and the biotinylated RBDs were produced in 25 mL cultures of Expi293F Cells (ThermoFisher Scientific) grown in suspension using Expi293 Expression Medium (ThermoFisher Scientific) at 37°C in a humidified 5% CO_2 incubator rotating at 130 rpm. Cells grown to a density of 3 million cells per mL were transfected using the ExpiFectamine 293 Transfection Kit (ThermoFisher Scientific) and cultivated for four days. Proteins were purified from clarified supernatants using a nickel HisTrap HP affinity column (Cytiva) and washed with ten column volumes of 20 mM imidazole, 25 mM sodium phosphate pH 8.0, and 300 mM NaCl before elution with a gradient of 500 mM imidazole. Proteins were buffer exchanged into 20 mM sodium phosphate pH 8 and 100 mM NaCl and concentrated using 30 kDa or 10 kDa centrifugal filters (Amicon Ultra, MilliporeSigma) for hACE2 and the biotinylated RBDs, respectively, before being flash frozen.

The SARS-CoV-2 S ectodomains were produced in 100 mL cultures of Expi293F Cells (ThermoFisher Scientific) grown in suspension using Expi293 Expression Medium (ThermoFisher Scientific) at 37°C in a humidified 8% CO_2 incubator rotating at 130 rpm. Cells grown to a density of 2.5 million cells per mL were transfected using the ExpiFectamine 293 Transfection Kit (ThermoFisher Scientific) and cultivated for four days at which point the supernatant was harvested. S ectodomains were purified from clarified supernatants using a Cobalt affinity column (Cytiva, HiTrap TALON crude), washing with 20 column volumes of 20 mM Tris-HCl pH 8.0 and 150 mM NaCl and eluted with a gradient of 600 mM imidazole. The S ectodomain was then concentrated using a 100 kDa centrifugal filter (Amicon Ultra 0.5 mL centrifugal filters, MilliporeSigma), residual imidazole was washed away by consecutive dilutions in the centrifugal filter unit with 20 mM Tris-HCl pH 8.0 and 150 mM NaCl, and finally concentrated to 2 mg/mL and flash frozen.

Production of VSV pseudovirus:

SARS-CoV-2 D614G, B.1.617.1 (mutations T95I, G142D, E154K, L452R, E484Q, D614G, P681R and Q1071H), B.1.617.2 (mutations T19, G142D, E156G, 157-158del, T478K, D950N) and B.1.617.2+ (B.1.617.2 mutations plus K417N) pseudotypes were prepared similarly as previously described (*52*). Briefly, HEK-293T cells seeded in poly-D-lysine coated 100 mm dishes at ~75 % confluency were washed five times with Opti-MEM and co-transfected with Lipofectamine 2000 (Life Technologies) with 24 μg of the respective S glycoprotein plasmids. After 5 h at 37°C, media supplemented with 20% FBS and 2% PenStrep was added. After 20 hours, cells were washed five times with DMEM and cells were transduced with VSVΔG-luc (*40*) and incubated at 37°C. After 2 h, infected cells were washed an additional five times with DMEM prior to adding media supplemented with anti-VSV-G antibody (I1-mouse hybridoma supernatant diluted 1:25, from CRL-2700, ATCC) to reduce parental background. After 18-24 h, the supernatant was harvested and clarified by low-speed centrifugation at 2,500 g for 10 min. The supernatant was then filtered (0.45 μm) and concentrated 10 times using a 30 kDa cut off membrane. The pseudotypes were then aliquoted and frozen at -80 °C.

VSV neutralization:

To evaluate neutralization of D614G, B.1.617.1, B.1.617.2 and B.1.617.2 + pseudotypes by sera of individuals immunized with Pfizer/BioNtech BNT162b2, Moderna mRNA1273 and Janssen Ad26.COV2.S, HEK-293T cells expressing hACE2 (*41*) in DMEM supplemented with 10% FBS and 1% PenStrep were seeded at 20,000 cells per well into clear bottom, white manually poly-D-lysine coated 96 well plates and incubated at 37°C.

The following day, an additional half-area, 96-well plate was prepared with eight 3-fold serial dilutions of sera from Pfizer/BioNtech BNT162b2, Moderna mRNA1273 or Janssen Ad26.COV2.S vaccine recipients. An equal volume of each 1:25 DMEM diluted D614G, B.1.617.1, B.1.617.2 and B.1.617.2+ pseudovirus were then added to the half-area plate. The mixture was then incubated at room temperature for 30 minutes.

At this stage, excess DMEM was removed from the cells and 40 μL from each well (containing sera and pseudovirus) was transferred to the 96-well plate seeded with HEK-293T cells expressing hACE2 and incubated at 37°C for 2 h. After 2 h, an additional 40 μL of DMEM supplemented with 20% FBS and 2% PenStrep was added to the cells.

After 20 h, 40 μL of One-Glo-EX substrate (Promega) was added to each well and incubated on a plate shaker in the dark for 5 min. The plates were immediately read on a Biotek plate reader. Measurements were done in duplicate with biological replicates. Relative luciferase units were plotted and normalized in Prism (GraphPad): cells alone without pseudovirus was defined as 0 % infection, and cells with virus only (no sera) was defined as 100 % infection. Prism

(GraphPad) nonlinear regression with "[inhibitor] versus normalized response with a variable slope" was used to determine IC50 values from curve fits. Means of these duplicates were compared against G614 by two-way ANOVA (Dunnet's test) using GraphPad PRISM software (version 9.2.0).

Western Blot

Undiluted pseudoviruses were added to 4X SDS-PAGE loading buffer. Samples were run on a 4%–15% gradient Tris-Glycine Gel (BioRad) and transferred to PVDF membranes. An anti-S_2 SARS-CoV-2 S polyclonal primary antibody (1:1,500 dilution,Invitrogen PA5-114534) and an Alexa Fluor 680-conjugated goat anti-rabbit secondary antibody (1:20,000 dilution, Jackson Laboratory 111-625-144) were used for Western-blotting. A LI-COR processor was used to develop images.

ELISA

For ELISA experiments with NTD-targeted mAbs, 384-well Maxisorp plates (ThermoFisher Scientific 464718) were coated overnight at 4°C with 2 μg/mL of S glycoprotein in 20mM HEPES pH 8 and 150mM NaCl. Plates were slapped dry and blocked with Blocker Casein in TBS (ThermoFisher Scientific 37532) for one hour at 37°C. Plates were slapped dry and mAbs were serially diluted 1:5 in TBST with an initial concentration of 50 μg/ml. Plates were left for one hour at 37°C and washed 4x with TBST, then 1:5000 Goat anti-Human (ThermoFisher Scientific A18817) was added. Plates were left for one hour at 37°C and washed 4x with TBST, and then TMB Microwell Peroxidase (Seracare 5120-0083) was added. The reaction was quenched after 4 minutes with 1 N HCl and the A450 of each well was read using a BioTek plate reader.

For ELISA experiments with RBDs, 384-well Maxisorp plates (ThermoFisher Scientific 464718) were coated overnight at 4°C with 4 μg/mL of hACE2-His in 20mM Sodium Phosphate pH 8 and 100mM NaCl. Plates were slapped dry and blocked with Blocker Casein in TBS (ThermoFisher Scientific 37532) for one hour at 37°C. Plates were slapped dry and wild-type RBD, B.1.1.7 RBD, B.1.351 RBD, B.1.617.1 RBD, B.1.617.2 RBD, and B.1.617.2+ RBD were serially diluted 1:3 in TBST with an initial concentration of 1542nM. Plates were left for one hour at 37°C, then washed 4x with TBST using a 405 TS Microplate Washer (BioTek) followed by addition of 2 μg/mL S309 mAb (*15*). Plates were left for one hour at 37°C and washed 4x with TBST, then 1:5000 Goat anti-Human (ThermoFisher Scientific A18817) was added. TMB Microwell Peroxidase (Seracare 5120-0083) was added after another hour at 37°C and 4x wash with TBST. The reaction was quenched after 1-2 minutes with 1 N HCl and the A450 of each well was read using a BioTek plate reader.

CryoEM sample preparation and data collection

50 µL of 2 mg/mL SARS-CoV-2 S B.1.617.1 ectodomain was incubated with 29 µl 3.4 mg/ml S309 Fab and 2.2 µL of 67 mg/mL S2L20 Fab in 150 mM NaCl and 20 mM Tris-HCl pH 8 for 30 min at 37°C. Alternatively, 50 µL of 2 mg/mL SARS-CoV-2 S B.1.617.2 ectodomain was incubated with 34 µl 2.9 mg/ml S2M11 Fab in 150 mM NaCl and 20 mM Tris-HCl pH 8 for 10 min at 37°C, and then 2.2 µL of 67 mg/mL S2L20 Fab was added for 20 min at 37°C. Alternatively, 50 µL of 2 mg/mL SARS-CoV-2 S B.1.617.1 ectodomain was incubated with 3.6 µl 28 mg/ml S2X303 Fab in 150 mM NaCl and 20 mM Tris-HCl pH 8 for 30 min at 37°C.

Unbound Fab was then washed away with six consecutive dilutions in 400 µL of 20 mM Tris-HCl pH 8.0 and 150 mM NaCl over a 100 kDa centrifugal filter (Amicon Ultra 0.5 mL centrifugal filters, MilliporeSigma). The complex was concentrated to 3.6 mg/mL and 3 µL was immediately applied onto a freshly glow discharged 2.0/2.0 UltraFoil grid (*84*) (200 mesh), plunge frozen using a vitrobot MarkIV (ThermoFisher Scientific) using a blot force of −1 and 6.5 s blot time at 100% humidity and 23°C.

Data were acquired using the Leginon software (*85*) to control a FEI Titan Krios transmission electron microscope equipped with a Gatan K3 direct detector and operated at 300 kV with a Gatan Quantum GIF energy filter. The dose rate was adjusted to 3.75 counts/super-resolution pixel/s, and each movie was acquired in 75 frames of 40 ms with a pixel size of 0.843 Å and a defocus range comprised between −0.2 and −2.0 µm.

CryoEM data processing

Movie frame alignment, estimation of the microscope contrast-transfer function parameters, particle picking and extraction (with a downsampled pixel size of 1.686 Å and box size of 256 pixels^2) were carried out using Warp (*86*). Reference-free 2D classification was performed using cryoSPARC (*87*) to select well-defined particle images. 3D classification with 50 iterations each (angular sampling 7.5˚for 25 iterations and 1.8˚with local search for 25 iterations) were carried out using Relion without imposing symmetry. 3D refinements were carried out using non-uniform refinement in cryoSPARC (*88*) before particle images were subjected to Bayesian polishing using Relion (*89*) during which particles were re-extracted with a box size of 512 Å at a pixel size of 0.843 Å. Next, 86 optics groups were defined based on the beamtilt angle used for data collection. Another round of non-uniform refinement in cryoSPARC was then performed concurrently with global and per-particle defocus refinement. For focused classification, particles were symmetry-expanded in Relion (*90*, *91*), and the particles were 3D classified in Relion without alignment using a mask that encompasses part of the NTD and the S2L20 VH/VL region, or the RBD and the S309 VH/VL region. Particles in well-formed 3D classes were then used for local refinement in cryoSPARC. Reported resolutions are based on the gold-standard Fourier shell correlation of 0.143 criterion and Fourier shell correlation curves were corrected for the effects of soft masking by high-resolution noise substitution (*92*, *93*).

CryoEM model building and analysis

UCSF Chimera (*94*) and Coot (*95*) were used to fit atomic models of S2M11, S2L20, S309, and SARS-CoV- 2 S (PDB 7LXY, 7N8I, 7R6W) into the cryo-EM maps. The model was then refined and rebuilt into the map using Coot (*95*), Rosetta (*96*, *97*), Phenix (*98*), and ISOLDE (*99*). Model validation and analysis used MolProbity (*100*), EMringer (*101*), Phenix (*98*) and Privateer (*102*). Figures were generated using UCSF ChimeraX (*103*).

ACE2 binding measurements using Biolayer interferometry

His-avi-tagged wildtype, B.1.1.7 (N501Y) B.1.617.1 (L452R, E484Q), B.1.617.2 (L452R, T478K), or B.1.617.2+ (K417N, L452R, T478K) RBD were biotinylated and immobilized at 5 ng/μL in undiluted 10X kinetics buffer (Pall) to SA sensors that were pre-hydrated in water for at least 10 minutes and then equilibrated into 10X Kinetics Buffer (Pall). The RBDs were loaded to a level of 1nm total shift. The loaded tips were then dipped into a dilution series of monomeric ACE2-his in 10X Kinetics Buffer (Pall) starting at 1000 or 5000 nM for 300 seconds prior to 300 seconds dissociation in 10X Kinetics buffer for kinetics determination. The data were baseline subtracted and the plots fitted using the Pall FortéBio/Sartorius analysis software (v.12.0). Data were plotted in Graphpad Prism (v.9.0.2). These experiments were done side-by-side with two separate RBD protein preparations and two separate ACE2 preparations and a representative experiment is shown.

ACE2 binding measurements using surface plasmon resonance

Measurements were performed using a Biacore T200 instrument. The Cytiva Biotin CAPture Kit, Series S, was used for surface capture of biotinylated RBDs. Running buffer was HBS-EP+ pH 7.4 (Cytiva) and measurements were performed at 25℃. Experiments were performed with a 3-fold dilution series of monomeric hACE2: 200 nM, 67 nM, 22 nM, 7.4 nM for the first experiment, and 300, 100, 33, 11, 3.7 nM for the second experiment (except for B.1.617.2+, which was 600, 200, 66.7, 22, 7.4 nM for the second experiment). Association was 300 s and dissociation was 450 s. Data were double reference-subtracted and fit to a 1:1 binding model using Biacore Evaluation software.

Pfizer - BNT162b2

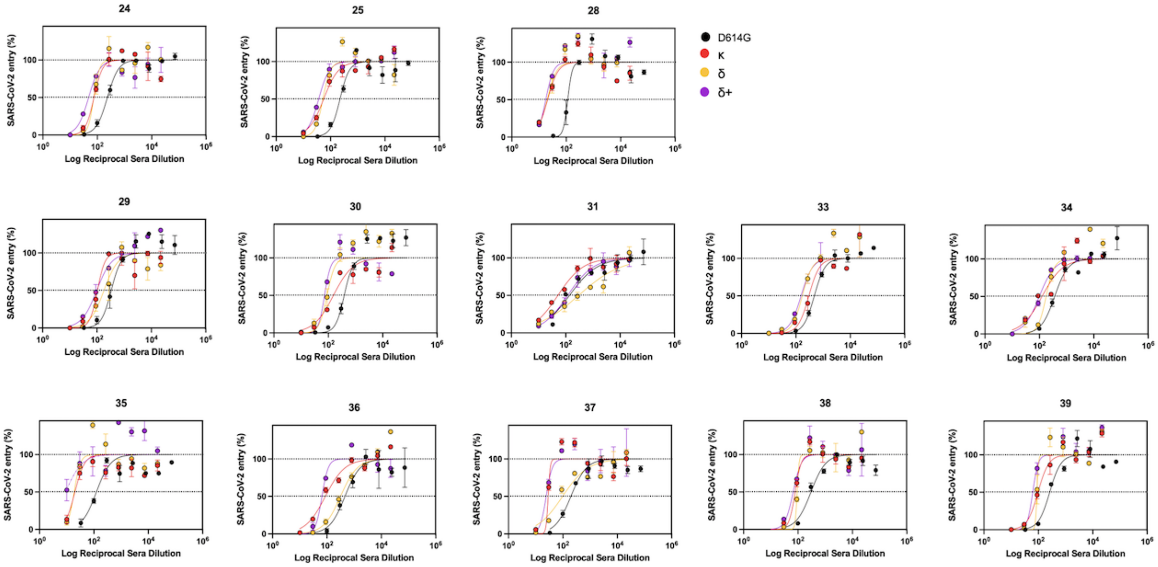

Moderna - mRNA-1273

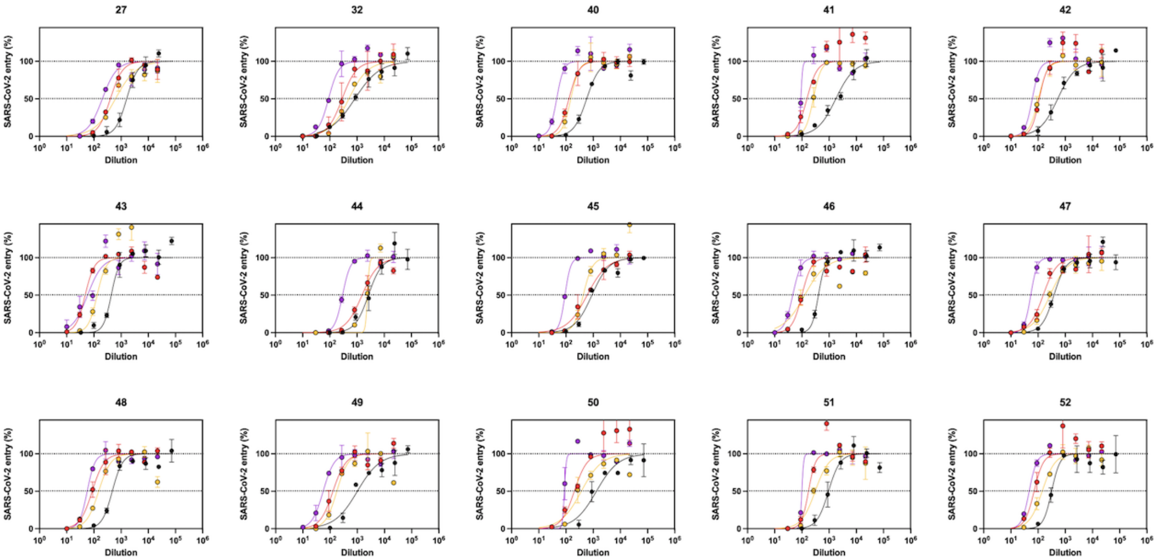

Janssen - Ad26.COV2.S

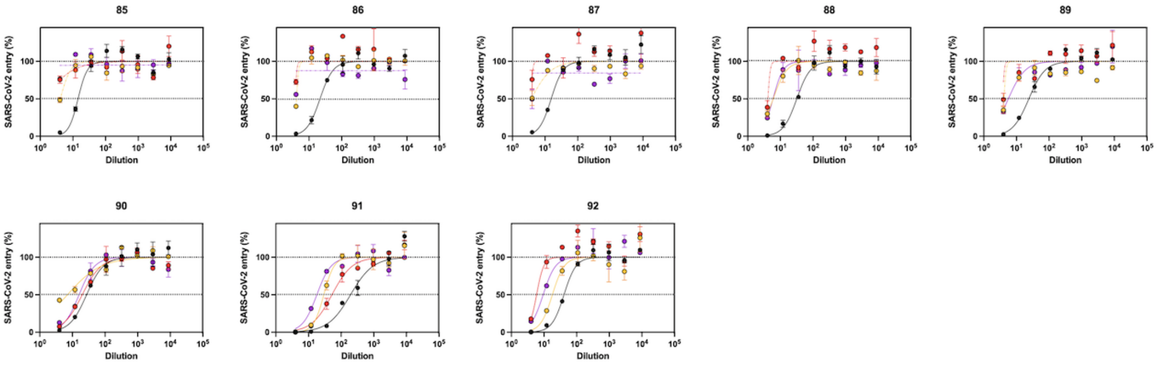

Figure S1: Plasma neutralizing activity of vaccinated humans. Related to Fig. 1A-C. Normalized neutralization data points and fits obtained with plasma from individuals vaccinated with Pfizer/BioNtech BNT162b2, Moderna mRNA-1273, or Janssen Ad26.COV2.S against G614, κ, δ, and δ+ VSV pseudovirus.

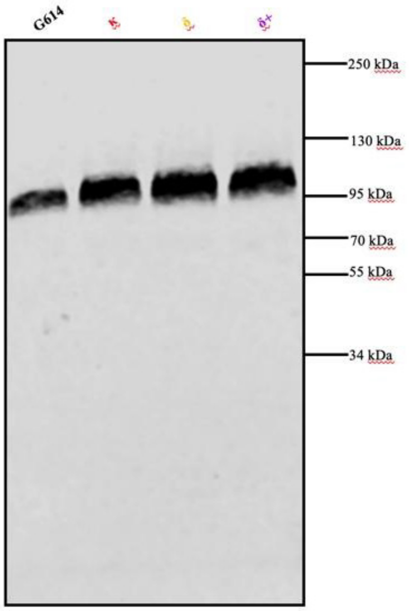

Figure S2: Western blot comparing S of pseudotypes used for neutralization. VSV pseudotype G614, κ, δ, and δ+ S visualized by western blotting showing relative amounts of S used in the neutralization assays.

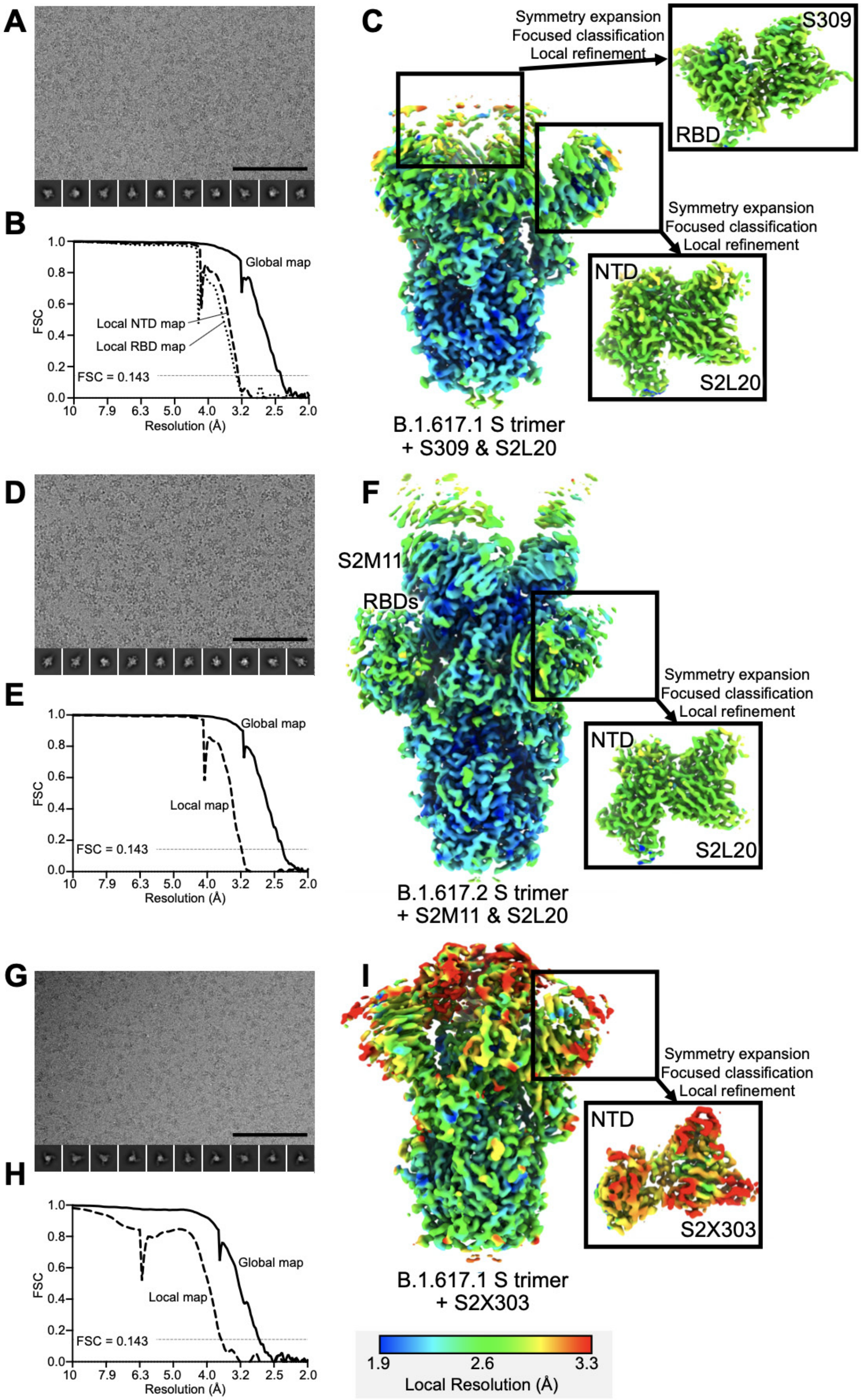
A
B
1.0
0.8
0.6
0.4
0.2
0.0
FSC
Global map
Local NTD map
Local RBD map
FSC = 0.143
10
7.9
6.3
5.0
4.0
3.2
2.5
2.0
Resolution (Å)
C
Symmetry expansion
Focused classification
Local refinement
S309
RBD
Symmetry expansion
Focused classification
Local refinement
NTD
S2L20
B.1.617.1 S trimer
+ S309 & S2L20
D
E
Global map
Local map
FSC = 0.143
Resolution (Å)
F
S2M11
RBDs
Symmetry expansion
Focused classification
Local refinement
NTD
S2L20
B.1.617.2 S trimer
+ S2M11 & S2L20
G
H
Global map
Local map
FSC = 0.143
Resolution (Å)
I
Symmetry expansion
Focused classification
Local refinement
NTD
S2X303
B.1.617.1 S trimer
+ S2X303
1.9
2.6
3.3
Local Resolution (Å)

Fig. S3. CryoEM data processing of SARS-CoV-2 B.1.617.1 S ectodomain trimer bound to S2L20 and S309 (A-C), B.1.617.2 S ectodomain trimer bound to S2L20 and S2M11 (D-F), and B.1.617.1 S ectodomain trimer bound to S2X303 (G-I). (A, D, and G) Representative electron micrograph (bottom right, scale bar: 100 nm) and 2D class averages (bottom) are shown for the indicated particles embedded in vitreous ice. **(B, E, and H)** Gold-standard Fourier shell correlation curves with the 0.143 cutoff indicated by a horizontal dashed line. **(C, F, and I)** Unsharpened maps colored by local resolution calculated using cryoSPARC for the whole reconstruction and the locally refined reconstructions of NTD- or RBD-bound Fab variable domains (insets).

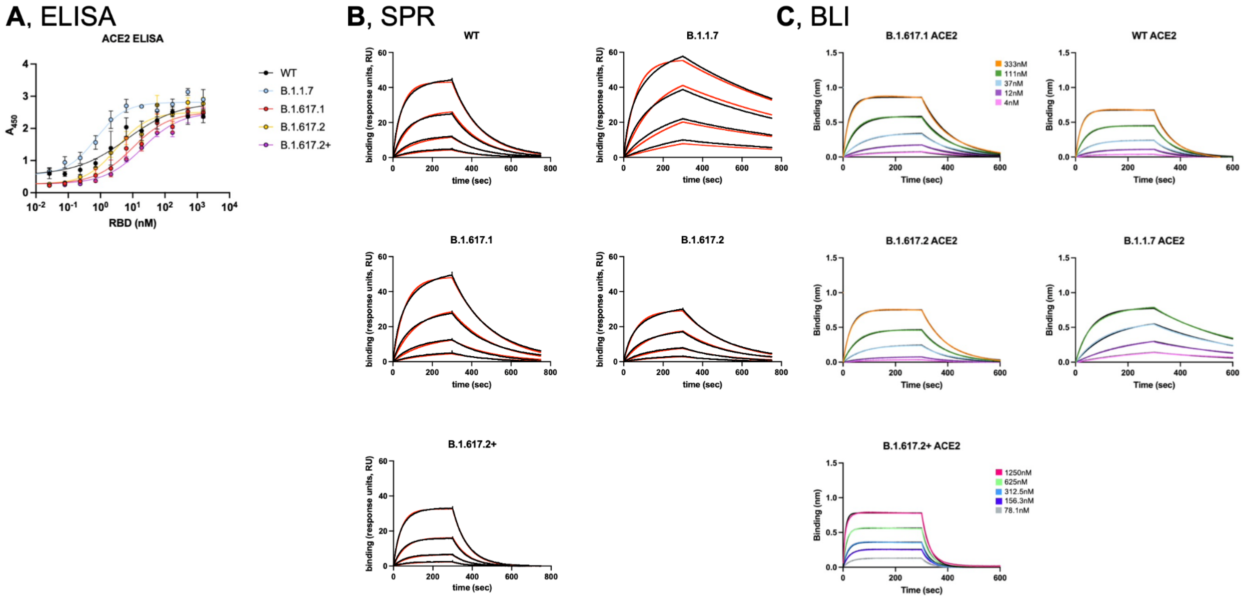

Figure S4: Binding kinetics of RBDs to ACE2 using ELISA, SPR, and BLI. (**A)** Binding of the SARS-CoV-2 wildtype, B.1.1.7 (α), B.1.351 (β), B.1.617.1 (κ), B.1.617.2 (δ), and B.1.617.2+ (δ+) RBDs to immobilized human ACE2 ectodomain analyzed by ELISA. (**B**) Binding of human ACE2 to the immobilized SARS-CoV-2 wildtype, B.1.1.7 (α), B.1.617.1 (κ), B.1.617.2 (δ), and B.1.617.2+ (δ+) RBDs analyzed by SPR. (**C**) Binding human ACE2 to the immobilized SARS-CoV-2 wildtype, B.1.1.7 (α), B.1.617.1 (κ), B.1.617.2 (δ), and B.1.617.2+ (δ+) RBDs analyzed by BLI. Binding rate constants and affinities are shown in Table S4.

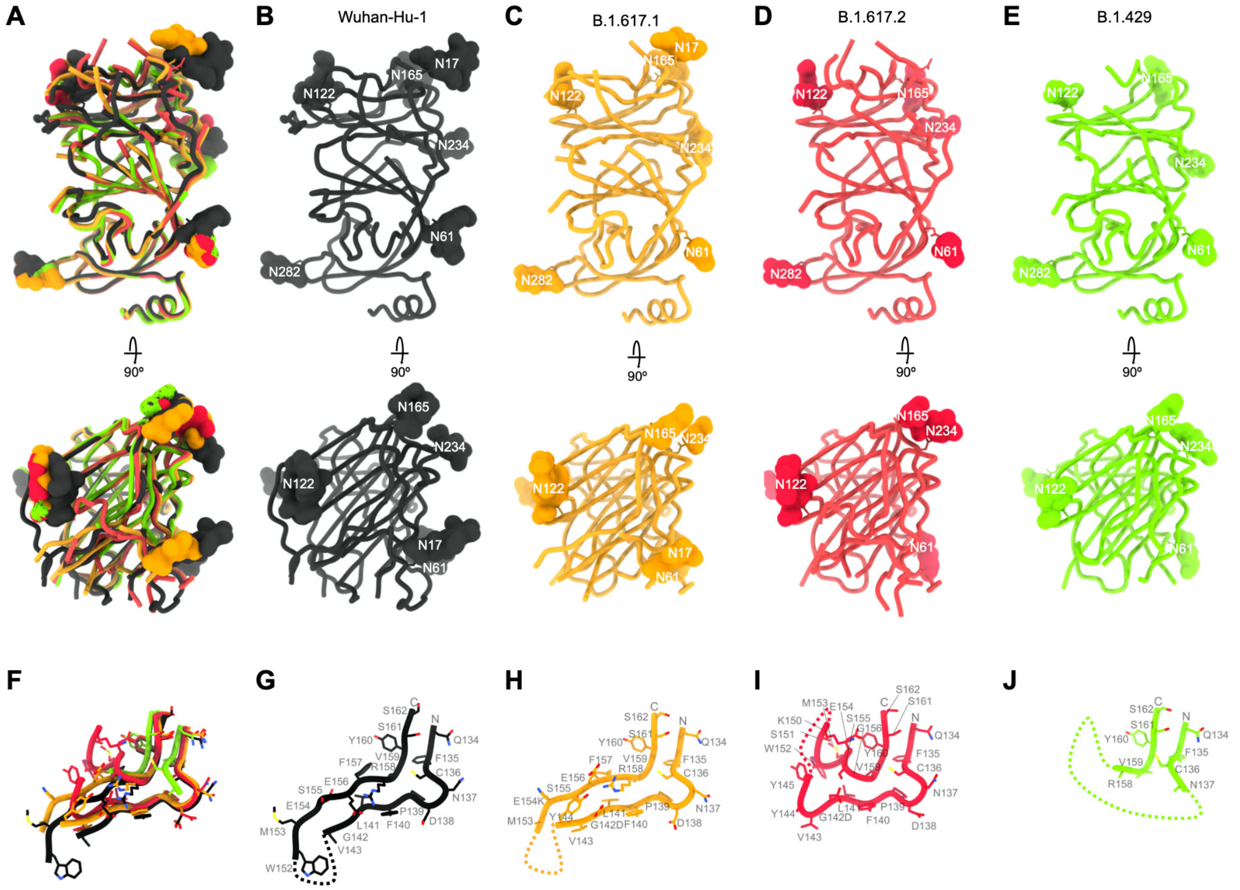

Figure S5. Comparison of variant NTDs. A-E, Top and side-view ribbon diagrams of overlaid NTDs (**A**), the Wuhan-Hu-1 NTD (**B**, PDB 7K43 (*19*)), the B.1.617.1 NTD (**C**), the B.1.617.2 NTD (**D**), and the B.1.429 NTD (**E**, PDB 7N8I (*52*)) rendered in the same orientation. Glycans are shown as surface representations. **F-J**, zoomed in view of NTD supersite β-hairpin residues Q134-S162 of overlaid NTDs (**F**) from the Wuhan-Hu-1 NTD (**G**, PDB 7K43), the B.1.617.1 NTD (**H**), the B.1.617.2 NTD (**I**), and the B.1.429 NTD (**J**, PDB 7N8I).

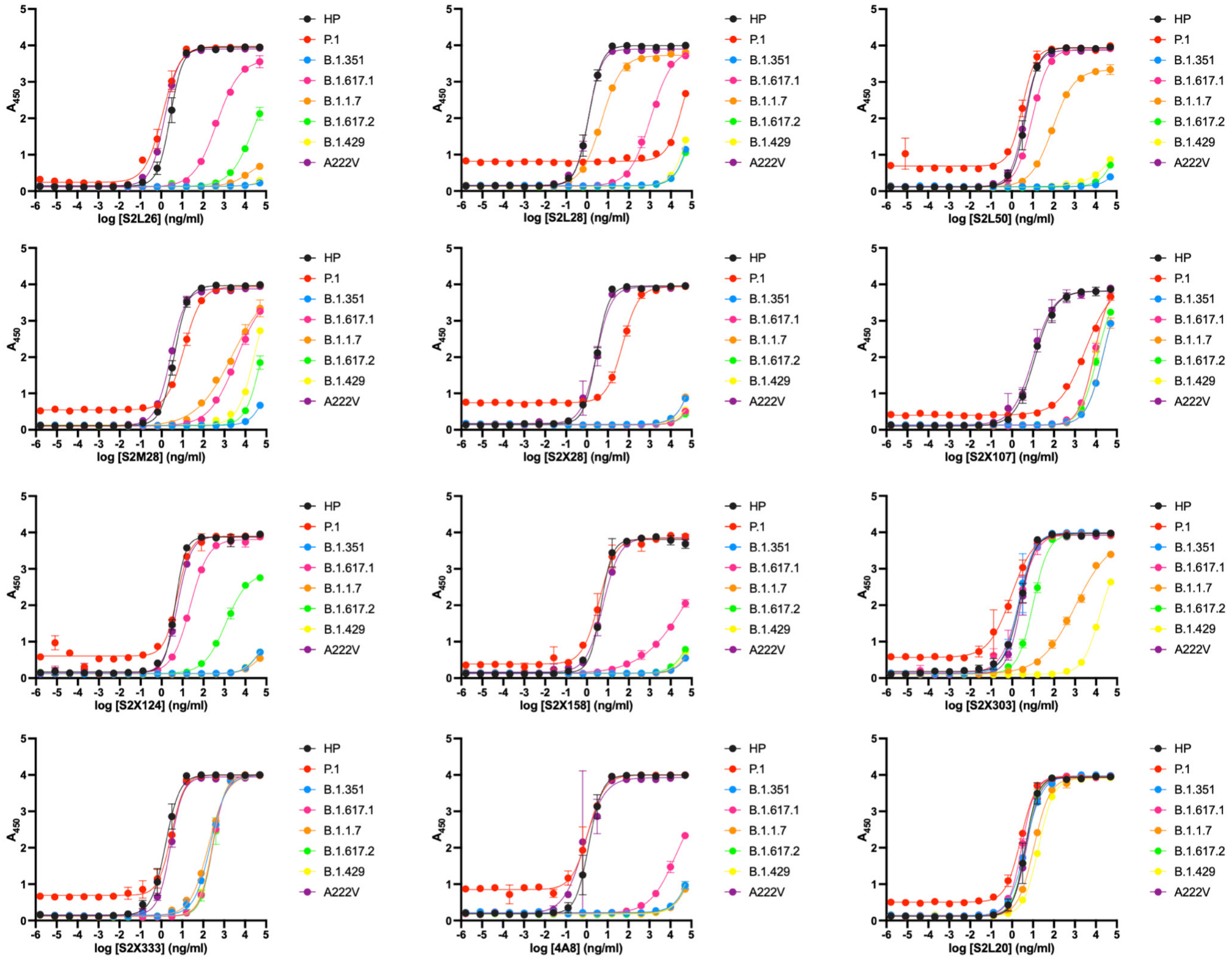

Fig. S6. Effect of SARS-CoV-2 variant mutations on NTD-targeted mAb binding. Binding of a panel of 11 neutralizing (antigenic site i) and 1 non-neutralizing (antigenic site iv, S2L20) NTD-specific mAbs to recombinant SARS-CoV-2 variant ectodomains analyzed by ELISA.

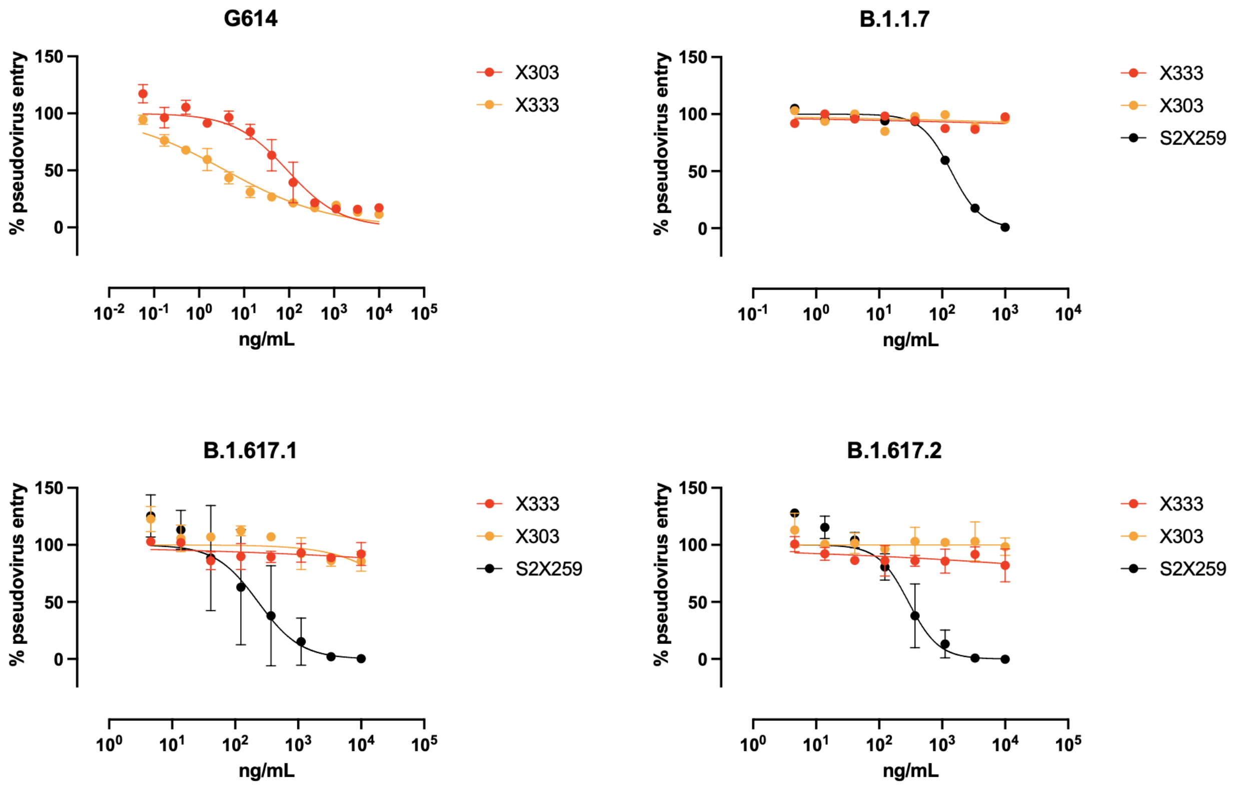

Figure S7: S2X303 neutralizes G614 S but not variant S pseudoviruses. Concentration-dependent mAb-mediated neutralization of G614 (**A**), B.1.1.7 (α) (**B**), B.1.617.2 (δ) (**C**), and B.1.617.1 (**κ**) (**D**) S VSV pseudoviruses. The NTD-specific mAb S2X333 (*23*) and the RBD-specific mAb S2X259 (*16*) were included as controls

Table S1: Summary of mutations present in select SARS-CoV-2 S variants.

WHO	PANGO	Signal Peptide	NTD	RBD	S1/S2	S2	Sequences (Sept 30 2021)
Alpha (α)	B.1.1.7	-	69-70del, Y144del	N501Y	A570D, D614G, P681H	T716I, S982A, D1118H	1,101,148
Beta (β)	B.1.351	-	L18F, D80A, D215G, 242-244del, R246I	K417N, E484K, N501Y	D614G	A701V	36,181
Gamma (γ)	P.1	-	L18F, T20N, P26S, D138Y, R190S	K417T, E484K, N501Y	D614G, H655Y	T1027I, V1176F	92,182
Delta (δ)	B.1.617.2	-	T19R, G142D, E156G, 157-158del	L452R, T478K	D614G, P681R	D950N	1,415,761
Delta+ (δ+)	B.1.617.2+	-	T19R, G142D, E156G, 157-158del	K417N, L452R, T478K	D614G, P681R	D950N	62
	AY.1	-	T19R, G142D, E156G, 157-158del, W258L	K417N, L452R, T478K	D614G, P681R	D950N	1,536
	AY.2	-	T19R, V70F, G142D, E156G, 157-158del, A222V	K417N, L452R, T478K	D614G, P681R	D950N	2,368
Epsilon (ε)	B.1.427/ B.1.429	S13I	W152C	L452R	D614G	-	60,008
Iota (ι)	B.1.526	L5F	T95I, D253G	E484K	D614G	-	40,820
Kappa (κ)	B.1.617.1	-	T95I, G142D, E154K	L452R, E484Q	D614G, P681R	Q1071H	6,806

Table S2: Demographics of vaccinated individuals.

Study ID	Age	Vaccine Type	Days post second vaccine	M/F	Race	Ethnicity
23	60	Pfizer	11	M	White	Not Hispanic or Latino
24	65	Pfizer	10	M	White	Not Hispanic or Latino
25	55	Pfizer	18	M	White	Not Hispanic or Latino
26	42	Pfizer	9	F	White	Not Hispanic or Latino
27	66	Moderna	8	F	White	Not Hispanic or Latino
28	63	Pfizer	10	M	White	Not Hispanic or Latino
29	27	Pfizer	8	F	White	Not Hispanic or Latino

30	38	Pfizer	8	F	Asian	Not Hispanic or Latino
31	37	Pfizer	21	F	Black	Not Hispanic or Latino
32	36	Moderna	7	M	White	Not Hispanic or Latino
33	62	Pfizer	15	M	Pacific Islander	Not Hispanic or Latino
34	54	Pfizer	14	F	White	Not Hispanic or Latino
35	60	Pfizer	14	F	White	Not Hispanic or Latino
36	32	Pfizer	13	F	White	Not Hispanic or Latino
37	52	Pfizer	11	M	White	Not Hispanic or Latino
38	61	Pfizer	9	M	White	Not Hispanic or Latino

39	32	Pfizer	22	F	White	Not Hispanic or Latino
40	40	Moderna	20	M	White	Not Hispanic or Latino
41	64	Moderna	16	M	White	Not Hispanic or Latino
42	34	Moderna	23	F	Asian	Not Hispanic or Latino
43	22	Moderna	20	F	White	Not Hispanic or Latino
44	24	Moderna	18	F	White	Not Hispanic or Latino
45	35	Moderna	20	M	White	Not Hispanic or Latino
46	40	Moderna	24	M	White	Not Hispanic or Latino
47	55	Moderna	20	M	White	Not Hispanic or Latino
48	25	Moderna	22	M	White and Asian	Not Hispanic or Latino

49	26	Moderna	18	F	White	Not Hispanic or Latino
50	36	Moderna	27	F	Asian	Not Hispanic or Latino
51	53	Moderna	20	F	White	Not Hispanic or Latino
52	47	Moderna	21	M	White	Not Hispanic or Latino

Participant ID	Age of enrollee (years)	Sex	Hispanic or Latino?	Race/ethnicity	Which vaccine did you receive?	Days since first dose:
85H	29	Male	No	White	Johnson & Johnson	102
86H	26	Female	Prefer not to say	Asian	Johnson & Johnson	90
88H	28	Female	No	White	Johnson & Johnson	100
87H	30	Female	No	Asian,White	Johnson & Johnson	100

89H	31	Male	No	White	Johnson & Johnson	102
90H	38	Male	No	Native Hawaiian or other Pacific Islander	Johnson & Johnson	132
91H	26	Female	Yes	White	Johnson & Johnson	105
92H	33	Female	No	Asian	Johnson & Johnson	102

Table S3. Cryo-EM data collection, refinement and validation statistics.

	**B.1.617.2 S + S2M11 + S2L20**		**B.1.617.1 S + S2X303**		**B.1.617.1 S + S309 + S2L20**		
**Data collection and processing**							
Magnification (nominal)	130,000		130,000		130,000		
Voltage (kV)	300		300		300		
Electron exposure (e^{-}/Å^2)	63		63		63		
Defocus range (μm)	0.3-2.0		0.3-2.0		0.3-2.0		
Pixel size (Å)	0.843		0.843		0.843		
Processing Type	Global	Local (NTD)	Global	Local (NTD)	Global	Local (RBD)	Local (NTD)
Symmetry imposed	C3	C1	C3	C1	C3	C1	C1
Initial particle images (no.)	955,640	1,631,004	151,568	227,916	454,334	734,817	734,817
Final particle images (no.)	543,668	281,779	75,972	80,186	244,939	277,827	217,346
Map resolution (Å)	2.4	3.1	2.8	3.6	2.4	3.3	3.2
FSC threshold	0.143	0.143	0.143	0.143	0.143	0.143	0.143
**Refinement**							
Initial model used (PDB code)	7N8H	7N8I	7LXY	7LXY	7N8H	7R6W	7N8I
Model resolution (Å)	2.3	3.1	2.7	3.5	2.4	3.2	3.1
FSC threshold	0.143	0.143	0.143	0.143	0.143	0.143	0.143
Map sharpening *B* factor (Å^2)	78	120	70	94	71	118	112
Model composition							
Nonhydrogen atoms	34,470	3850	25,947	3735	30,000	3444	3830
Protein residues	4434	477	3783	488	4443	433	473
Glycan residues	51	6	57	9	51	7	7
B factors (Å^2)							
Protein	18	14	50	33	44	16	17
Glycans	17	20	47	38	26	23	24
R.m.s. deviations							
Bond lengths (Å)	0.01	0.01	0.01	0.01	0.01	0.01	0.1
Bond angles (°)	1.0	1.1	1.0	1.0	1.1	1.0	1.0

**Validation**							
MolProbity score	0.7	0.8	0.8	1.0	0.9	0.8	0.7
Clashscore	0.7	1.1	1.0	1.1	1.3	0.9	0.4
Rotamer outliers (%)	0.2	0.2	0.2	0.0	0.0	0.0	0.5
Ramachandran plot							
Favored (%)	98	98	98	97	98	98	98
Allowed (%)	2	2	2	3	2	2	2
Outliers (%)	0	0	0	0	0	0	0
EMRinger score	4.8	4.7	4.0	4.1	4.8	4.5	5.2
**Data Availability**							
EMDB	EMD-25263	EMD-25264	EMD-25268	EMD-25269	EMD-25265	EMD-25266	EMD-25267
PDB	7SO9	7SOA	7SOE	7SOF	7SOB	7SOC	7SOD

Table S4: Binding kinetics of RBD to ACE2.

**ELISA**	**EC50 (nM) ± SEM**		
**WT**	5 ± 1		
**B.1.1.7**	0.48 ± 0.02		
**B.1.617.1**	9 ± 1		
**B.1.617.2**	5 ± 2		
**B.1.617.2+**	40 ± 20		
**SPR**	**K_D (nM) ± SEM**	**k_{on} ($M^{-1}s^{-1}$)**	**k_{off} (s^{-1})**
**WT**	78 ± 8	7.7×10^4	6.7×10^{-3}
**B.1.1.7**	15.0 ± 0.4	7.5×10^4	1.2×10^{-3}
**B.1.617.1**	71 ± 3	6.0×10^4	4.8×10^{-3}
**B.1.617.2**	63 ± 3	5.9×10^4	4.3×10^{-3}
**B.1.617.2+**	183 ± 4	6.6×10^4	1.3×10^{-2}
**BLI**	**K_D (nM) ± SEM**	**k_{on} ($M^{-1}s^{-1}$)**	**k_{off} (s^{-1})**
**WT**	147 ± 3	9.5×10^4	1.4×10^{-2}
**B.1.1.7**	26 ± 4	1.3×10^5	2.8×10^{-3}
**B.1.617.1**	88 ± 1	1.0×10^5	9.1×10^{-3}
**B.1.617.2**	180 ± 30	7.4×10^4	1.1×10^{-2}
**B.1.617.2+**	520 ± 60	6.9×10^4	3.2×10^{-2}

References and Notes

1. S. Cele, I. Gazy, L. Jackson, S.-H. Hwa, H. Tegally, G. Lustig, J. Giandhari, S. Pillay, E. Wilkinson, Y. Naidoo, F. Karim, Y. Ganga, K. Khan, M. Bernstein, A. B. Balazs, B. I. Gosnell, W. Hanekom, M. S. Moosa, R. J. Lessells, T. de Oliveira, A. Sigal; Network for Genomic Surveillance in South Africa; COMMIT-KZN Team, Escape of SARS-CoV-2 501Y.V2 from neutralization by convalescent plasma. *Nature* **593**, 142–146 (2021). doi:10.1038/s41586-021-03471-w Medline

2. H. Tegally, E. Wilkinson, M. Giovanetti, A. Iranzadeh, V. Fonseca, J. Giandhari, D. Doolabh, S. Pillay, E. J. San, N. Msomi, K. Mlisana, A. von Gottberg, S. Walaza, M. Allam, A. Ismail, T. Mohale, A. J. Glass, S. Engelbrecht, G. Van Zyl, W. Preiser, F. Petruccione, A. Sigal, D. Hardie, G. Marais, N. Y. Hsiao, S. Korsman, M.-A. Davies, L. Tyers, I. Mudau, D. York, C. Maslo, D. Goedhals, S. Abrahams, O. Laguda-Akingba, A. Alisoltani-Dehkordi, A. Godzik, C. K. Wibmer, B. T. Sewell, J. Lourenço, L. C. J. Alcantara, S. L. Kosakovsky Pond, S. Weaver, D. Martin, R. J. Lessells, J. N. Bhiman, C. Williamson, T. de Oliveira, Detection of a SARS-CoV-2 variant of concern in South Africa. *Nature* **592**, 438–443 (2021). doi:10.1038/s41586-021-03402-9 Medline

3. C. K. Wibmer, F. Ayres, T. Hermanus, M. Madzivhandila, P. Kgagudi, B. Oosthuysen, B. E. Lambson, T. de Oliveira, M. Vermeulen, K. van der Berg, T. Rossouw, M. Boswell, V. Ueckermann, S. Meiring, A. von Gottberg, C. Cohen, L. Morris, J. N. Bhiman, P. L. Moore, SARS-CoV-2 501Y.V2 escapes neutralization by South African COVID-19 donor plasma. *Nat. Med.* **27**, 622–625 (2021). doi:10.1038/s41591-021-01285-x Medline

4. D. A. Collier, A. De Marco, I. A. T. M. Ferreira, B. Meng, R. P. Datir, A. C. Walls, S. A. Kemp, J. Bassi, D. Pinto, C. Silacci-Fregni, S. Bianchi, M. A. Tortorici, J. Bowen, K. Culap, S. Jaconi, E. Cameroni, G. Snell, M. S. Pizzuto, A. F. Pellanda, C. Garzoni, A. Riva, A. Elmer, N. Kingston, B. Graves, L. E. McCoy, K. G. C. Smith, J. R. Bradley, N. Temperton, L. Ceron-Gutierrez, G. Barcenas-Morales, W. Harvey, H. W. Virgin, A. Lanzavecchia, L. Piccoli, R. Doffinger, M. Wills, D. Veesler, D. Corti, R. K. Gupta; CITIID-NIHR BioResource COVID-19 Collaboration; COVID-19 Genomics UK (COG-UK) Consortium, Sensitivity of SARS-CoV-2 B.1.1.7 to mRNA vaccine-elicited antibodies. *Nature* **593**, 136–141 (2021). doi:10.1038/s41586-021-03412-7 Medline

5. A. C. Walls, Y. J. Park, M. A. Tortorici, A. Wall, A. T. McGuire, D. Veesler, Structure, function, and antigenicity of the SARS-CoV-2 spike glycoprotein. *Cell* **181**, 281–292.e6 (2020). doi:10.1016/j.cell.2020.02.058 Medline

6. D. Wrapp, N. Wang, K. S. Corbett, J. A. Goldsmith, C. L. Hsieh, O. Abiona, B. S. Graham, J. S. McLellan, Cryo-EM structure of the 2019-nCoV spike in the prefusion conformation. *Science* **367**, 1260–1263 (2020). doi:10.1126/science.abb2507 Medline

7. M. Hoffmann, H. Kleine-Weber, S. Schroeder, N. Krüger, T. Herrler, S. Erichsen, T. S. Schiergens, G. Herrler, N. H. Wu, A. Nitsche, M. A. Müller, C. Drosten, S. Pöhlmann, SARS-CoV-2 cell entry depends on ACE2 and TMPRSS2 and is blocked by a clinically proven protease inhibitor. *Cell* **181**, 271–280.e8 (2020). doi:10.1016/j.cell.2020.02.052 Medline

8. M. Hoffmann, H. Kleine-Weber, S. Pöhlmann, A multibasic cleavage site in the spike protein of SARS-CoV-2 is essential for infection of human lung cells. *Mol. Cell* **78**, 779–784.e5 (2020). doi:10.1016/j.molcel.2020.04.022 Medline

9. M. Letko, A. Marzi, V. Munster, Functional assessment of cell entry and receptor usage for SARS-CoV-2 and other lineage B betacoronaviruses. *Nat. Microbiol.* **5**, 562–569 (2020). doi:10.1038/s41564-020-0688-y Medline

10. P. Zhou, X. L. Yang, X. G. Wang, B. Hu, L. Zhang, W. Zhang, H. R. Si, Y. Zhu, B. Li, C. L. Huang, H. D. Chen, J. Chen, Y. Luo, H. Guo, R. D. Jiang, M. Q. Liu, Y. Chen, X. R. Shen, X. Wang, X. S. Zheng, K. Zhao, Q. J. Chen, F. Deng, L. L. Liu, B. Yan, F. X. Zhan, Y. Y. Wang, G. F. Xiao, Z. L. Shi, A pneumonia outbreak associated with a new coronavirus of probable bat origin. *Nature* **579**, 270–273 (2020). doi:10.1038/s41586-020-2012-7 Medline

11. S. Wang, Z. Qiu, Y. Hou, X. Deng, W. Xu, T. Zheng, P. Wu, S. Xie, W. Bian, C. Zhang, Z. Sun, K. Liu, C. Shan, A. Lin, S. Jiang, Y. Xie, Q. Zhou, L. Lu, J. Huang, X. Li, AXL is a candidate receptor for SARS-CoV-2 that promotes infection of pulmonary and bronchial epithelial cells. *Cell Res.* **31**, 126–140 (2021). doi:10.1038/s41422-020-00460-y Medline

12. W. T. Soh, Y. Liu, E. E. Nakayama, C. Ono, S. Torii, H. Nakagami, Y. Matsuura, T. Shioda, H. Arase, The N-terminal domain of spike glycoprotein mediates SARS-CoV-2 infection by associating with L-SIGN and DC-SIGN. bioRxiv 2020.11.05.369264 [Preprint] (2020). https://doi.org/10.1101/2020.11.05.369264.

13. F. A. Lempp, L. B. Soriaga, M. Montiel-Ruiz, F. Benigni, J. Noack, Y.-J. Park, S. Bianchi, A. C. Walls, J. E. Bowen, J. Zhou, H. Kaiser, A. Joshi, M. Agostini, M. Meury, E. Dellota Jr., S. Jaconi, E. Cameroni, J. Martinez-Picado, J. Vergara-Alert, N. Izquierdo-Useros, H. W. Virgin, A. Lanzavecchia, D. Veesler, L. A. Purcell, A. Telenti, D. Corti, Lectins enhance SARS-CoV-2 infection and influence neutralizing antibodies. *Nature* **598**, 342–347 (2021). doi:10.1038/s41586-021-03925-1 Medline

14. A. C. Walls, M. A. Tortorici, J. Snijder, X. Xiong, B. J. Bosch, F. A. Rey, D. Veesler, Tectonic conformational changes of a coronavirus spike glycoprotein promote membrane fusion. *Proc. Natl. Acad. Sci. U.S.A.* **114**, 11157–11162 (2017). doi:10.1073/pnas.1708727114 Medline

15. D. Pinto, Y. J. Park, M. Beltramello, A. C. Walls, M. A. Tortorici, S. Bianchi, S. Jaconi, K. Culap, F. Zatta, A. De Marco, A. Peter, B. Guarino, R. Spreafico, E. Cameroni, J. B. Case, R. E. Chen, C. Havenar-Daughton, G. Snell, A. Telenti, H. W. Virgin, A. Lanzavecchia, M. S. Diamond, K. Fink, D. Veesler, D. Corti, Cross-neutralization of SARS-CoV-2 by a human monoclonal SARS-CoV antibody. *Nature* **583**, 290–295 (2020). doi:10.1038/s41586-020-2349-y Medline

16. M. A. Tortorici, N. Czudnochowski, T. N. Starr, R. Marzi, A. C. Walls, F. Zatta, J. E. Bowen, S. Jaconi, J. Di Iulio, Z. Wang, A. De Marco, S. K. Zepeda, D. Pinto, Z. Liu, M. Beltramello, I. Bartha, M. P. Housley, F. A. Lempp, L. E. Rosen, E. Dellota Jr., H. Kaiser, M. Montiel-Ruiz, J. Zhou, A. Addetia, B. Guarino, K. Culap, N. Sprugasci, C. Saliba, E. Vetti, I. Giacchetto-Sasselli, C. S. Fregni, R. Abdelnabi, S. C. Foo, C. Havenar-Daughton, M. A. Schmid, F. Benigni, E. Cameroni, J. Neyts, A. Telenti, H. W. Virgin, S. P. J. Whelan, G. Snell, J. D. Bloom, D. Corti, D. Veesler, M. S. Pizzuto, Broad

sarbecovirus neutralization by a human monoclonal antibody. *Nature* **597**, 103–108 (2021). doi:10.1038/s41586-021-03817-4 Medline

17. T. N. Starr, N. Czudnochowski, Z. Liu, F. Zatta, Y.-J. Park, A. Addetia, D. Pinto, M. Beltramello, P. Hernandez, A. J. Greaney, R. Marzi, W. G. Glass, I. Zhang, A. S. Dingens, J. E. Bowen, M. A. Tortorici, A. C. Walls, J. A. Wojcechowskyj, A. De Marco, L. E. Rosen, J. Zhou, M. Montiel-Ruiz, H. Kaiser, J. R. Dillen, H. Tucker, J. Bassi, C. Silacci-Fregni, M. P. Housley, J. di Iulio, G. Lombardo, M. Agostini, N. Sprugasci, K. Culap, S. Jaconi, M. Meury, E. Dellota Jr., R. Abdelnabi, S. C. Foo, E. Cameroni, S. Stumpf, T. I. Croll, J. C. Nix, C. Havenar-Daughton, L. Piccoli, F. Benigni, J. Neyts, A. Telenti, F. A. Lempp, M. S. Pizzuto, J. D. Chodera, C. M. Hebner, H. W. Virgin, S. P. J. Whelan, D. Veesler, D. Corti, J. D. Bloom, G. Snell, SARS-CoV-2 RBD antibodies that maximize breadth and resistance to escape. *Nature* **597**, 97–102 (2021). doi:10.1038/s41586-021-03807-6 Medline

18. L. Piccoli, Y. J. Park, M. A. Tortorici, N. Czudnochowski, A. C. Walls, M. Beltramello, C. Silacci-Fregni, D. Pinto, L. E. Rosen, J. E. Bowen, O. J. Acton, S. Jaconi, B. Guarino, A. Minola, F. Zatta, N. Sprugasci, J. Bassi, A. Peter, A. De Marco, J. C. Nix, F. Mele, S. Jovic, B. F. Rodriguez, S. V. Gupta, F. Jin, G. Piumatti, G. Lo Presti, A. F. Pellanda, M. Biggiogero, M. Tarkowski, M. S. Pizzuto, E. Cameroni, C. Havenar-Daughton, M. Smithey, D. Hong, V. Lepori, E. Albanese, A. Ceschi, E. Bernasconi, L. Elzi, P. Ferrari, C. Garzoni, A. Riva, G. Snell, F. Sallusto, K. Fink, H. W. Virgin, A. Lanzavecchia, D. Corti, D. Veesler, Mapping neutralizing and immunodominant sites on the SARS-CoV-2 spike receptor-binding domain by structure-guided high-resolution serology. *Cell* **183**, 1024–1042.e21 (2020). doi:10.1016/j.cell.2020.09.037 Medline

19. M. A. Tortorici, M. Beltramello, F. A. Lempp, D. Pinto, H. V. Dang, L. E. Rosen, M. McCallum, J. Bowen, A. Minola, S. Jaconi, F. Zatta, A. De Marco, B. Guarino, S. Bianchi, E. J. Lauron, H. Tucker, J. Zhou, A. Peter, C. Havenar-Daughton, J. A. Wojcechowskyj, J. B. Case, R. E. Chen, H. Kaiser, M. Montiel-Ruiz, M. Meury, N. Czudnochowski, R. Spreafico, J. Dillen, C. Ng, N. Sprugasci, K. Culap, F. Benigni, R. Abdelnabi, S. C. Foo, M. A. Schmid, E. Cameroni, A. Riva, A. Gabrieli, M. Galli, M. S. Pizzuto, J. Neyts, M. S. Diamond, H. W. Virgin, G. Snell, D. Corti, K. Fink, D. Veesler, Ultrapotent human antibodies protect against SARS-CoV-2 challenge via multiple mechanisms. *Science* **370**, 950–957 (2020). doi:10.1126/science.abe3354 Medline

20. B. E. Jones, P. L. Brown-Augsburger, K. S. Corbett, K. Westendorf, J. Davies, T. P. Cujec, C. M. Wiethoff, J. L. Blackbourne, B. A. Heinz, D. Foster, R. E. Higgs, D. Balasubramaniam, L. Wang, Y. Zhang, E. S. Yang, R. Bidshahri, L. Kraft, Y. Hwang, S. Žentelis, K. R. Jepson, R. Goya, M. A. Smith, D. W. Collins, S. J. Hinshaw, S. A. Tycho, D. Pellacani, P. Xiang, K. Muthuraman, S. Sobhanifar, M. H. Piper, F. J. Triana, J. Hendle, A. Pustilnik, A. C. Adams, S. J. Berens, R. S. Baric, D. R. Martinez, R. W. Cross, T. W. Geisbert, V. Borisevich, O. Abiona, H. M. Belli, M. de Vries, A. Mohamed, M. Dittmann, M. I. Samanovic, M. J. Mulligan, J. A. Goldsmith, C.-L. Hsieh, N. V. Johnson, D. Wrapp, J. S. McLellan, B. C. Barnhart, B. S. Graham, J. R. Mascola, C. L. Hansen, E. Falconer, The neutralizing antibody, LY-CoV555, protects against SARS-CoV-2 infection in nonhuman primates. *Sci. Transl. Med.* **13**, eabf1906 (2021). doi:10.1126/scitranslmed.abf1906 Medline

21. C. A. Jette, A. A. Cohen, P. N. P. Gnanapragasam, F. Muecksch, Y. E. Lee, K. E. Huey-Tubman, F. Schmidt, T. Hatziioannou, P. D. Bieniasz, M. C. Nussenzweig, A. P. West Jr., J. R. Keeffe, P. J. Bjorkman, C. O. Barnes, Broad cross-reactivity across sarbecoviruses exhibited by a subset of COVID-19 donor-derived neutralizing antibodies. *Cell Rep.* **36**, 109760 (2021). doi:10.1016/j.celrep.2021.109760 Medline

22. C. O. Barnes, C. A. Jette, M. E. Abernathy, K. A. Dam, S. R. Esswein, H. B. Gristick, A. G. Malyutin, N. G. Sharaf, K. E. Huey-Tubman, Y. E. Lee, D. F. Robbiani, M. C. Nussenzweig, A. P. West Jr., P. J. Bjorkman, SARS-CoV-2 neutralizing antibody structures inform therapeutic strategies. *Nature* **588**, 682–687 (2020). doi:10.1038/s41586-020-2852-1 Medline

23. M. McCallum, A. De Marco, F. A. Lempp, M. A. Tortorici, D. Pinto, A. C. Walls, M. Beltramello, A. Chen, Z. Liu, F. Zatta, S. Zepeda, J. di Iulio, J. E. Bowen, M. Montiel-Ruiz, J. Zhou, L. E. Rosen, S. Bianchi, B. Guarino, C. S. Fregni, R. Abdelnabi, S.-Y. C. Foo, P. W. Rothlauf, L.-M. Bloyet, F. Benigni, E. Cameroni, J. Neyts, A. Riva, G. Snell, A. Telenti, S. P. J. Whelan, H. W. Virgin, D. Corti, M. S. Pizzuto, D. Veesler, N-terminal domain antigenic mapping reveals a site of vulnerability for SARS-CoV-2. *Cell* **184**, 2332–2347.e16 (2021). doi:10.1016/j.cell.2021.03.028 Medline

24. G. Cerutti, Y. Guo, T. Zhou, J. Gorman, M. Lee, M. Rapp, E. R. Reddem, J. Yu, F. Bahna, J. Bimela, Y. Huang, P. S. Katsamba, L. Liu, M. S. Nair, R. Rawi, A. S. Olia, P. Wang, B. Zhang, G.-Y. Chuang, D. D. Ho, Z. Sheng, P. D. Kwong, L. Shapiro, Potent SARS-CoV-2 neutralizing antibodies directed against spike N-terminal domain target a single supersite. *Cell Host Microbe* **29**, 819–833.e7 (2021). doi:10.1016/j.chom.2021.03.005 Medline

25. X. Chi, R. Yan, J. Zhang, G. Zhang, Y. Zhang, M. Hao, Z. Zhang, P. Fan, Y. Dong, Y. Yang, Z. Chen, Y. Guo, J. Zhang, Y. Li, X. Song, Y. Chen, L. Xia, L. Fu, L. Hou, J. Xu, C. Yu, J. Li, Q. Zhou, W. Chen, A neutralizing human antibody binds to the N-terminal domain of the Spike protein of SARS-CoV-2. *Science* **369**, 650–655 (2020). doi:10.1126/science.abc6952 Medline

26. N. Suryadevara, S. Shrihari, P. Gilchuk, L. A. VanBlargan, E. Binshtein, S. J. Zost, R. S. Nargi, R. E. Sutton, E. S. Winkler, E. C. Chen, M. E. Fouch, E. Davidson, B. J. Doranz, R. E. Chen, P.-Y. Shi, R. H. Carnahan, L. B. Thackray, M. S. Diamond, J. E. Crowe Jr., Neutralizing and protective human monoclonal antibodies recognizing the N-terminal domain of the SARS-CoV-2 spike protein. *Cell* **184**, 2316–2331.e15 (2021). doi:10.1016/j.cell.2021.03.029 Medline

27. M. M. Sauer, M. A. Tortorici, Y.-J. Park, A. C. Walls, L. Homad, O. J. Acton, J. E. Bowen, C. Wang, X. Xiong, W. de van der Schueren, J. Quispe, B. G. Hoffstrom, B. J. Bosch, A. T. McGuire, D. Veesler, Structural basis for broad coronavirus neutralization. *Nat. Struct. Mol. Biol.* **28**, 478–486 (2021). doi:10.1038/s41594-021-00596-4 Medline

28. C. Wang, R. van Haperen, J. Gutiérrez-Álvarez, W. Li, N. M. A. Okba, I. Albulescu, I. Widjaja, B. van Dieren, R. Fernandez-Delgado, I. Sola, D. L. Hurdiss, O. Daramola, F. Grosveld, F. J. M. van Kuppeveld, B. L. Haagmans, L. Enjuanes, D. Drabek, B.-J. Bosch, A conserved immunogenic and vulnerable site on the coronavirus spike protein

delineated by cross-reactive monoclonal antibodies. *Nat. Commun.* **12**, 1715 (2021). doi:10.1038/s41467-021-21968-w Medline

29. P. Zhou, M. Yuan, G. Song, N. Beutler, N. Shaabani, D. Huang, W.-T. He, X. Zhu, S. Callaghan, P. Yong, F. Anzanello, L. Peng, J. Ricketts, M. Parren, E. Garcia, S. A. Rawlings, D. M. Smith, D. Nemazee, J. R. Teijaro, T. F. Rogers, I. A. Wilson, D. R. Burton, R. Andrabi, A protective broadly cross-reactive human antibody defines a conserved site of vulnerability on beta-coronavirus spikes. bioRxiv 2021.03.30.437769 [Preprint] (2021). https://doi.org/10.1101/2021.03.30.437769.

30. D. Pinto, M. M. Sauer, N. Czudnochowski, J. S. Low, M. A. Tortorici, M. P. Housley, J. Noack, A. C. Walls, J. E. Bowen, B. Guarino, L. E. Rosen, J. di Iulio, J. Jerak, H. Kaiser, S. Islam, S. Jaconi, N. Sprugasci, K. Culap, R. Abdelnabi, C. Foo, L. Coelmont, I. Bartha, S. Bianchi, C. Silacci-Fregni, J. Bassi, R. Marzi, E. Vetti, A. Cassotta, A. Ceschi, P. Ferrari, P. E. Cippà, O. Giannini, S. Ceruti, C. Garzoni, A. Riva, F. Benigni, E. Cameroni, L. Piccoli, M. S. Pizzuto, M. Smithey, D. Hong, A. Telenti, F. A. Lempp, J. Neyts, C. Havenar-Daughton, A. Lanzavecchia, F. Sallusto, G. Snell, H. W. Virgin, M. Beltramello, D. Corti, D. Veesler, Broad betacoronavirus neutralization by a stem helix–specific human antibody. *Science* **373**, 1109–1116 (2021). doi:10.1126/science.abj3321 Medline

31. G. Song, W.-T. He, S. Callaghan, F. Anzanello, D. Huang, J. Ricketts, J. L. Torres, N. Beutler, L. Peng, S. Vargas, J. Cassell, M. Parren, L. Yang, C. Ignacio, D. M. Smith, J. E. Voss, D. Nemazee, A. B. Ward, T. Rogers, D. R. Burton, R. Andrabi, Cross-reactive serum and memory B-cell responses to spike protein in SARS-CoV-2 and endemic coronavirus infection. *Nat. Commun.* **12**, 2938 (2021). doi:10.1038/s41467-021-23074-3 Medline

32. P. S. Arunachalam, A. C. Walls, N. Golden, C. Atyeo, S. Fischinger, C. Li, P. Aye, M. J. Navarro, L. Lai, V. V. Edara, K. Röltgen, K. Rogers, L. Shirreff, D. E. Ferrell, S. Wrenn, D. Pettie, J. C. Kraft, M. C. Miranda, E. Kepl, C. Sydeman, N. Brunette, M. Murphy, B. Fiala, L. Carter, A. G. White, M. Trisal, C.-L. Hsieh, K. Russell-Lodrigue, C. Monjure, J. Dufour, S. Spencer, L. Doyle-Meyers, R. P. Bohm, N. J. Maness, C. Roy, J. A. Plante, K. S. Plante, A. Zhu, M. J. Gorman, S. Shin, X. Shen, J. Fontenot, S. Gupta, D. T. O’Hagan, R. Van Der Most, R. Rappuoli, R. L. Coffman, D. Novack, J. S. McLellan, S. Subramaniam, D. Montefiori, S. D. Boyd, J. L. Flynn, G. Alter, F. Villinger, H. Kleanthous, J. Rappaport, M. S. Suthar, N. P. King, D. Veesler, B. Pulendran, Adjuvanting a subunit COVID-19 vaccine to induce protective immunity. *Nature* **594**, 253–258 (2021). doi:10.1038/s41586-021-03530-2 Medline

33. K. McMahan, J. Yu, N. B. Mercado, C. Loos, L. H. Tostanoski, A. Chandrashekar, J. Liu, L. Peter, C. Atyeo, A. Zhu, E. A. Bondzie, G. Dagotto, M. S. Gebre, C. Jacob-Dolan, Z. Li, F. Nampanya, S. Patel, L. Pessaint, A. Van Ry, K. Blade, J. Yalley-Ogunro, M. Cabus, R. Brown, A. Cook, E. Teow, H. Andersen, M. G. Lewis, D. A. Lauffenburger, G. Alter, D. H. Barouch, Correlates of protection against SARS-CoV-2 in rhesus macaques. *Nature* **590**, 630–634 (2021). doi:10.1038/s41586-020-03041-6 Medline

34. D. S. Khoury, D. Cromer, A. Reynaldi, T. E. Schlub, A. K. Wheatley, J. A. Juno, K. Subbarao, S. J. Kent, J. A. Triccas, M. P. Davenport, Neutralizing antibody levels are

highly predictive of immune protection from symptomatic SARS-CoV-2 infection. *Nat. Med.* **27**, 1205–1211 (2021). doi:10.1038/s41591-021-01377-8 Medline

35. K. S. Corbett, M. C. Nason, B. Flach, M. Gagne, S. O'Connell, T. S. Johnston, S. N. Shah, V. V. Edara, K. Floyd, L. Lai, C. McDanal, J. R. Francica, B. Flynn, K. Wu, A. Choi, M. Koch, O. M. Abiona, A. P. Werner, J. I. Moliva, S. F. Andrew, M. M. Donaldson, J. Fintzi, D. R. Flebbe, E. Lamb, A. T. Noe, S. T. Nurmukhambetova, S. J. Provost, A. Cook, A. Dodson, A. Faudree, J. Greenhouse, S. Kar, L. Pessaint, M. Porto, K. Steingrebe, D. Valentin, S. Zouantcha, K. W. Bock, M. Minai, B. M. Nagata, R. van de Wetering, S. Boyoglu-Barnum, K. Leung, W. Shi, E. S. Yang, Y. Zhang, J. M. Todd, L. Wang, G. S. Alvarado, H. Andersen, K. E. Foulds, D. K. Edwards, J. R. Mascola, I. N. Moore, M. G. Lewis, A. Carfi, D. Montefiori, M. S. Suthar, A. McDermott, M. Roederer, N. J. Sullivan, D. C. Douek, B. S. Graham, R. A. Seder, Immune correlates of protection by mRNA-1273 vaccine against SARS-CoV-2 in nonhuman primates. *Science* **373**, eabj0299 (2021). doi:10.1126/science.abj0299 Medline

36. S. Cherian, V. Potdar, S. Jadhav, P. Yadav, N. Gupta, M. Das, P. Rakshit, S. Singh, P. Abraham, S. Panda, SARS-CoV-2 spike mutations, L452R, T478K, E484Q and P681R, in the second wave of COVID-19 in Maharashtra, India. *Microorganisms* **9**, 1542 (2021). doi:10.3390/microorganisms9071542 Medline

37. P. Mlcochova, S. Kemp, M. S. Dhar, G. Papa, B. Meng, S. Mishra, C. Whittaker, T. Mellan, I. Ferreira, R. Datir, D. A. Collier, A. Albecka, S. Singh, R. Pandey, J. Brown, J. Zhou, N. Goonawardne, R. Marwal, M. Datta, S. Sengupta, K. Ponnusamy, V. S. Radhakrishnan, A. Abdullahi, O. Charles, P. Chattopadhyay, P. Devi, D. Caputo, T. Peacock, C. Wattal, N. Goel, A. Satwik, R. Vaishya, M. Agarwal, A. Mavousian, J. H. Lee, J. Bassi, C. Silacci-Fegni, C. Saliba, D. Pinto, T. Irie, I. Yoshida, W. L. Hamilton, K. Sato, L. James, D. Corti, L. Piccoli, S. Bhatt, S. Flaxman, W. S. Barclay, P. Rakshit, A. Agrawal, R. K. Gupta; The Indian SARS-CoV-2 Genomics Consortium (INSACOG); The CITIID-NIHR BioResource COVID-19 Collaboration; The Genotype to Phenotype Japan (G2P-Japan) Consortium, SARS-CoV-2 B.1.617.2 Delta variant replication, sensitivity to neutralising antibodies and vaccine breakthrough. bioRxiv 2021.05.08.443253 [Preprint] (2021). https://doi.org/10.1101/2021.05.08.443253.

38. A. J. Greaney, A. N. Loes, L. E. Gentles, K. H. D. Crawford, T. N. Starr, K. D. Malone, H. Y. Chu, J. D. Bloom, Antibodies elicited by mRNA-1273 vaccination bind more broadly to the receptor binding domain than do those from SARS-CoV-2 infection. *Sci. Transl. Med.* **13**, eabi9915 (2021). doi:10.1126/scitranslmed.abi9915 Medline

39. Z. Wang, F. Schmidt, Y. Weisblum, F. Muecksch, C. O. Barnes, S. Finkin, D. Schaefer-Babajew, M. Cipolla, C. Gaebler, J. A. Lieberman, T. Y. Oliveira, Z. Yang, M. E. Abernathy, K. E. Huey-Tubman, A. Hurley, M. Turroja, K. A. West, K. Gordon, K. G. Millard, V. Ramos, J. Da Silva, J. Xu, R. A. Colbert, R. Patel, J. Dizon, C. Unson-O'Brien, I. Shimeliovich, A. Gazumyan, M. Caskey, P. J. Bjorkman, R. Casellas, T. Hatziioannou, P. D. Bieniasz, M. C. Nussenzweig, mRNA vaccine-elicited antibodies to SARS-CoV-2 and circulating variants. *Nature* **592**, 616–622 (2021). doi:10.1038/s41586-021-03324-6 Medline

40. Y. Kaname, H. Tani, C. Kataoka, M. Shiokawa, S. Taguwa, T. Abe, K. Moriishi, T. Kinoshita, Y. Matsuura, Acquisition of complement resistance through incorporation of

CD55/decay-accelerating factor into viral particles bearing baculovirus GP64. *J. Virol.* **84**, 3210–3219 (2010). doi:10.1128/JVI.02519-09 Medline

41. K. H. D. Crawford, R. Eguia, A. S. Dingens, A. N. Loes, K. D. Malone, C. R. Wolf, H. Y. Chu, M. A. Tortorici, D. Veesler, M. Murphy, D. Pettie, N. P. King, A. B. Balazs, J. D. Bloom, Protocol and reagents for pseudotyping lentiviral particles with SARS-CoV-2 Spike protein for neutralization assays. *Viruses* **12**, 513 (2020). doi:10.3390/v12050513 Medline

42. A. C. Walls, M. C. Miranda, A. Schäfer, M. N. Pham, A. Greaney, P. S. Arunachalam, M.-J. Navarro, M. A. Tortorici, K. Rogers, M. A. O'Connor, L. Shirreff, D. E. Ferrell, J. Bowen, N. Brunette, E. Kepl, S. K. Zepeda, T. Starr, C.-L. Hsieh, B. Fiala, S. Wrenn, D. Pettie, C. Sydeman, K. R. Sprouse, M. Johnson, A. Blackstone, R. Ravichandran, C. Ogohara, L. Carter, S. W. Tilles, R. Rappuoli, S. R. Leist, D. R. Martinez, M. Clark, R. Tisch, D. T. O'Hagan, R. Van Der Most, W. C. Van Voorhis, D. Corti, J. S. McLellan, H. Kleanthous, T. P. Sheahan, K. D. Smith, D. H. Fuller, F. Villinger, J. Bloom, B. Pulendran, R. S. Baric, N. P. King, D. Veesler, Elicitation of broadly protective sarbecovirus immunity by receptor-binding domain nanoparticle vaccines. *Cell* **184**, 5432–5447.e16 (2021). doi:10.1016/j.cell.2021.09.015 Medline

43. R. Ukey, N. Bruiners, H. Mishra, P. K. Mishra, D. McCloskey, A. Onyuka, F. Chen, A. Pinter, D. Weiskopf, A. Sette, J. Roy, S. Gaur, M. L. Gennaro, Dichotomy between the humoral and cellular responses elicited by mRNA and adenoviral vector vaccines against SARS-CoV-2. medRxiv 2021.09.17.21263528 [Preprint] (2021). https://doi.org/10.1101/2021.09.17.21263528.

44. A. Sharma, G. Oda, M. Holodniy, COVID-19 vaccine breakthrough infections in Veterans Health Administration. medRxiv 2021.09.23.21263864 [Preprint] (2021). https://doi.org/10.1101/2021.09.23.21263864.

45. T. Kustin, N. Harel, U. Finkel, S. Perchik, S. Harari, M. Tahor, I. Caspi, R. Levy, M. Leshchinsky, S. Ken Dror, G. Bergerzon, H. Gadban, F. Gadban, E. Eliassian, O. Shimron, L. Saleh, H. Ben-Zvi, E. Keren Taraday, D. Amichay, A. Ben-Dor, D. Sagas, M. Strauss, Y. Shemer Avni, A. Huppert, E. Kepten, R. D. Balicer, D. Netzer, S. Ben-Shachar, A. Stern, Evidence for increased breakthrough rates of SARS-CoV-2 variants of concern in BNT162b2-mRNA-vaccinated individuals. *Nat. Med.* **27**, 1379–1384 (2021). doi:10.1038/s41591-021-01413-7 Medline

46. P. Y. Chia, S. W. Xiang Ong, C. J. Chiew, L. W. Ang, J.-M. Chavatte, T.-M. Mak, L. Cui, S. Kalimuddin, W. N. Chia, C. W. Tan, L. Y. Ann Chai, S. Y. Tan, S. Zheng, R. T. Pin Lin, L. Wang, Y.-S. Leo, V. J. Lee, D. C. Lye, B. E. Young, Virological and serological kinetics of SARS-CoV-2 Delta variant vaccine-breakthrough infections: a multi-center cohort study. medRxiv 2021.07.28.21261295 [Preprint] (2021). https://doi.org/10.1101/2021.07.28.21261295.

47. B. Li, A. Deng, K. Li, Y. Hu, Z. Li, Q. Xiong, Z. Liu, Q. Guo, L. Zou, H. Zhang, M. Zhang, F. Ouyang, J. Su, W. Su, J. Xu, H. Lin, J. Sun, J. Peng, H. Jiang, P. Zhou, T. Hu, M. Luo, Y. Zhang, H. Zheng, J. Xiao, T. Liu, R. Che, H. Zeng, Z. Zheng, Y. Huang, J. Yu, L. Yi, J. Wu, J. Chen, H. Zhong, X. Deng, M. Kang, O. G. Pybus, M. Hall, K. A. Lythgoe, Y. Li, J. Yuan, J. He, J. Lu, Viral infection and transmission in a large, well-traced outbreak

caused by the SARS-CoV-2 Delta variant. medRxiv 2021.07.07.21260122 [Preprint] (2021). https://doi.org/10.1101/2021.07.07.21260122.

48. P. Mlcochova, S. Kemp, M. S. Dhar, G. Papa, B. Meng, I. A. T. M. Ferreira, R. Datir, D. A. Collier, A. Albecka, S. Singh, R. Pandey, J. Brown, J. Zhou, N. Goonawardane, S. Mishra, C. Whittaker, T. Mellan, R. Marwal, M. Datta, S. Sengupta, K. Ponnusamy, V. S. Radhakrishnan, A. Abdullahi, O. Charles, P. Chattopadhyay, P. Devi, D. Caputo, T. Peacock, D. C. Wattal, N. Goel, A. Satwik, R. Vaishya, M. Agarwal, A. Mavousian, J. H. Lee, J. Bassi, C. Silacci-Fegni, C. Saliba, D. Pinto, T. Irie, I. Yoshida, W. L. Hamilton, K. Sato, S. Bhatt, S. Flaxman, L. C. James, D. Corti, L. Piccoli, W. S. Barclay, P. Rakshit, A. Agrawal, R. K. Gupta; Indian SARS-CoV-2 Genomics Consortium (INSACOG); Genotype to Phenotype Japan (G2P-Japan) Consortium; CITIID-NIHR BioResource COVID-19 Collaboration, SARS-CoV-2 B.1.617.2 Delta variant replication and immune evasion. *Nature* **599**, 114–119 (2021). doi:10.1038/s41586-021-03944-y

49. Y. Liu, J. Liu, B. A. Johnson, H. Xia, Z. Ku, C. Schindewolf, S. G. Widen, Z. An, S. C. Weaver, V. D. Menachery, X. Xie, P.-Y. Shi, Delta spike P681R mutation enhances SARS-CoV-2 fitness over Alpha variant. bioRxiv 2021.08.12.456173 [Preprint] (2021). https://doi.org/10.1101/2021.08.12.456173.

50. C. L. Hsieh, J. A. Goldsmith, J. M. Schaub, A. M. DiVenere, H. C. Kuo, K. Javanmardi, K. C. Le, D. Wrapp, A. G. Lee, Y. Liu, C. W. Chou, P. O. Byrne, C. K. Hjorth, N. V. Johnson, J. Ludes-Meyers, A. W. Nguyen, J. Park, N. Wang, D. Amengor, J. J. Lavinder, G. C. Ippolito, J. A. Maynard, I. J. Finkelstein, J. S. McLellan, Structure-based design of prefusion-stabilized SARS-CoV-2 spikes. *Science* **369**, 1501–1505 (2020). doi:10.1126/science.abd0826 Medline

51. M. McCallum, A. C. Walls, J. E. Bowen, D. Corti, D. Veesler, Structure-guided covalent stabilization of coronavirus spike glycoprotein trimers in the closed conformation. *Nat. Struct. Mol. Biol.* **27**, 942–949 (2020). doi:10.1038/s41594-020-0483-8 Medline

52. M. McCallum, J. Bassi, A. De Marco, A. Chen, A. C. Walls, J. Di Iulio, M. A. Tortorici, M.-J. Navarro, C. Silacci-Fregni, C. Saliba, K. R. Sprouse, M. Agostini, D. Pinto, K. Culap, S. Bianchi, S. Jaconi, E. Cameroni, J. E. Bowen, S. W. Tilles, M. S. Pizzuto, S. B. Guastalla, G. Bona, A. F. Pellanda, C. Garzoni, W. C. Van Voorhis, L. E. Rosen, G. Snell, A. Telenti, H. W. Virgin, L. Piccoli, D. Corti, D. Veesler, SARS-CoV-2 immune evasion by the B.1.427/B.1.429 variant of concern. *Science* **373**, 648–654 (2021). doi:10.1126/science.abi7994 Medline

53. E. Olmedillas, C. J. Mann, W. Peng, Y.-T. Wang, R. D. Avalos, D. Bedinger, K. Valentine, N. Shafee, S. L. Schendel, M. Yuan, G. Lang, R. Rouet, D. Christ, W. Jiang, I. A. Wilson, T. Germann, S. Shresta, J. Snijder, E. Ollmann Saphire, Structure-based design of a highly stable, covalently-linked SARS-CoV-2 spike trimer with improved structural properties and immunogenicity. bioRxiv 2021.05.06.441046 [Preprint] (2021). https://doi.org/10.1101/2021.05.06.441046.

54. C. O. Barnes, A. P. West Jr., K. E. Huey-Tubman, M. A. G. Hoffmann, N. G. Sharaf, P. R. Hoffman, N. Koranda, H. B. Gristick, C. Gaebler, F. Muecksch, J. C. C. Lorenzi, S. Finkin, T. Hägglöf, A. Hurley, K. G. Millard, Y. Weisblum, F. Schmidt, T. Hatziioannou, P. D. Bieniasz, M. Caskey, D. F. Robbiani, M. C. Nussenzweig, P. J. Bjorkman,

Structures of human antibodies bound to SARS-CoV-2 spike reveal common epitopes and recurrent features of antibodies. *Cell* **182**, 828–842.e16 (2020). doi:10.1016/j.cell.2020.06.025 Medline

55. S. J. Zost, P. Gilchuk, J. B. Case, E. Binshtein, R. E. Chen, J. P. Nkolola, A. Schäfer, J. X. Reidy, A. Trivette, R. S. Nargi, R. E. Sutton, N. Suryadevara, D. R. Martinez, L. E. Williamson, E. C. Chen, T. Jones, S. Day, L. Myers, A. O. Hassan, N. M. Kafai, E. S. Winkler, J. M. Fox, S. Shrihari, B. K. Mueller, J. Meiler, A. Chandrashekar, N. B. Mercado, J. J. Steinhardt, K. Ren, Y. M. Loo, N. L. Kallewaard, B. T. McCune, S. P. Keeler, M. J. Holtzman, D. H. Barouch, L. E. Gralinski, R. S. Baric, L. B. Thackray, M. S. Diamond, R. H. Carnahan, J. E. Crowe Jr., Potently neutralizing and protective human antibodies against SARS-CoV-2. *Nature* **584**, 443–449 (2020). doi:10.1038/s41586-020-2548-6 Medline

56. T. N. Starr, A. J. Greaney, A. S. Dingens, J. D. Bloom, Complete map of SARS-CoV-2 RBD mutations that escape the monoclonal antibody LY-CoV555 and its cocktail with LY-CoV016. *Cell Rep. Med.* **2**, 100255 (2021). doi:10.1016/j.xcrm.2021.100255 Medline

57. J. Hansen, A. Baum, K. E. Pascal, V. Russo, S. Giordano, E. Wloga, B. O. Fulton, Y. Yan, K. Koon, K. Patel, K. M. Chung, A. Hermann, E. Ullman, J. Cruz, A. Rafique, T. Huang, J. Fairhurst, C. Libertiny, M. Malbec, W. Y. Lee, R. Welsh, G. Farr, S. Pennington, D. Deshpande, J. Cheng, A. Watty, P. Bouffard, R. Babb, N. Levenkova, C. Chen, B. Zhang, A. Romero Hernandez, K. Saotome, Y. Zhou, M. Franklin, S. Sivapalasingam, D. C. Lye, S. Weston, J. Logue, R. Haupt, M. Frieman, G. Chen, W. Olson, A. J. Murphy, N. Stahl, G. D. Yancopoulos, C. A. Kyratsous, Studies in humanized mice and convalescent humans yield a SARS-CoV-2 antibody cocktail. *Science* **369**, 1010–1014 (2020). doi:10.1126/science.abd0827 Medline

58. T. Tada, H. Zhou, B. M. Dcosta, M. I. Samanovic, M. J. Mulligan, N. R. Landau, The spike proteins of SARS-CoV-2 B.1.617 and B.1.618 variants identified in India provide partial resistance to vaccine-elicited and therapeutic monoclonal antibodies. bioRxiv 2021.05.14.444076 [Preprint] (2021). https://doi.org/10.1101/2021.05.14.444076.

59. T. N. Starr, A. J. Greaney, A. Addetia, W. W. Hannon, M. C. Choudhary, A. S. Dingens, J. Z. Li, J. D. Bloom, Prospective mapping of viral mutations that escape antibodies used to treat COVID-19. *Science* **371**, 850–854 (2021). doi:10.1126/science.abf9302 Medline

60. A. L. Cathcart, C. Havenar-Daughton, F. A. Lempp, D. Ma, M. Schmid, M. L. Agostini, B. Guarino, J. Di iulio, L. Rosen, H. Tucker, J. Dillen, S. Subramanian, B. Sloan, S. Bianchi, J. Wojcechowskyj, J. Zhou, H. Kaiser, A. Chase, M. Montiel-Ruiz, N. Czudnochowski, E. Cameroni, S. Ledoux, C. Colas, L. Soriaga, A. Telenti, S. Hwang, G. Snell, H. W. Virgin, D. Corti, C. M. Hebner, The dual function monoclonal antibodies VIR-7831 and VIR-7832 demonstrate potent in vitro and in vivo activity against SARS-CoV-2. bioRxiv 2021.03.09.434607 [Preprint] (2021). https://doi.org/10.1101/2021.03.09.434607.

61. K. Wu, A. P. Werner, M. Koch, A. Choi, E. Narayanan, G. B. E. Stewart-Jones, T. Colpitts, H. Bennett, S. Boyoglu-Barnum, W. Shi, J. I. Moliva, N. J. Sullivan, B. S. Graham, A. Carfi, K. S. Corbett, R. A. Seder, D. K. Edwards, Serum neutralizing activity elicited by mRNA-1273 vaccine. *N. Engl. J. Med.* **384**, 1468–1470 (2021). doi:10.1056/NEJMc2102179 Medline

62. I. A. T. M. Ferreira, S. A. Kemp, R. Datir, A. Saito, B. Meng, P. Rakshit, A. Takaori-Kondo, Y. Kosugi, K. Uriu, I. Kimura, K. Shirakawa, A. Abdullahi, A. Agarwal, S. Ozono, K. Tokunaga, K. Sato, R. K. Gupta; CITIID-NIHR BioResource COVID-19 Collaboration, Indian SARS-CoV-2 Genomics Consortium; Genotype to Phenotype Japan (G2P-Japan) Consortium, SARS-CoV-2 B.1.617 Mutations L452R and E484Q Are Not Synergistic for Antibody Evasion. *J. Infect. Dis.* **224**, 989–994 (2021). doi:10.1093/infdis/jiab368 Medline

63. F. A. Lempp, L. Soriaga, M. Montiel-Ruiz, F. Benigni, J. Noack, Y.-J. Park, S. Bianchi, A. C. Walls, J. E. Bowen, J. Zhou, H. Kaiser, M. Agostini, M. Meury, E. Dellota Jr., S. Jaconi, E. Cameroni, H. W. Virgin, A. Lanzavecchia, D. Veesler, L. Purcell, A. Telenti, D. Corti, Membrane lectins enhance SARS-CoV-2 infection and influence the neutralizing activity of different classes of antibodies. bioRxiv 2021.04.03.438258 [Preprint] (2021). https://doi.org/10.1101/2021.04.03.438258.

64. T. N. Starr, A. J. Greaney, S. K. Hilton, D. Ellis, K. H. D. Crawford, A. S. Dingens, M. J. Navarro, J. E. Bowen, M. A. Tortorici, A. C. Walls, N. P. King, D. Veesler, J. D. Bloom, Deep mutational scanning of SARS-CoV-2 receptor binding domain reveals constraints on folding and ACE2 binding. *Cell* **182**, 1295–1310.e20 (2020). doi:10.1016/j.cell.2020.08.012 Medline

65. E. C. Thomson, L. E. Rosen, J. G. Shepherd, R. Spreafico, A. da Silva Filipe, J. A. Wojcechowskyj, C. Davis, L. Piccoli, D. J. Pascall, J. Dillen, S. Lytras, N. Czudnochowski, R. Shah, M. Meury, N. Jesudason, A. De Marco, K. Li, J. Bassi, A. O'Toole, D. Pinto, R. M. Colquhoun, K. Culap, B. Jackson, F. Zatta, A. Rambaut, S. Jaconi, V. B. Sreenu, J. Nix, I. Zhang, R. F. Jarrett, W. G. Glass, M. Beltramello, K. Nomikou, M. Pizzuto, L. Tong, E. Cameroni, T. I. Croll, N. Johnson, J. Di Iulio, A. Wickenhagen, A. Ceschi, A. M. Harbison, D. Mair, P. Ferrari, K. Smollett, F. Sallusto, S. Carmichael, C. Garzoni, J. Nichols, M. Galli, J. Hughes, A. Riva, A. Ho, M. Schiuma, M. G. Semple, P. J. M. Openshaw, E. Fadda, J. K. Baillie, J. D. Chodera, S. J. Rihn, S. J. Lycett, H. W. Virgin, A. Telenti, D. Corti, D. L. Robertson, G. Snell; ISARIC4C Investigators; COVID-19 Genomics UK (COG-UK) Consortium, Circulating SARS-CoV-2 spike N439K variants maintain fitness while evading antibody-mediated immunity. *Cell* **184**, 1171–1187.e20 (2021). doi:10.1016/j.cell.2021.01.037 Medline

66. M. Yuan, D. Huang, C. D. Lee, N. C. Wu, A. M. Jackson, X. Zhu, H. Liu, L. Peng, M. J. van Gils, R. W. Sanders, D. R. Burton, S. M. Reincke, H. Prüss, J. Kreye, D. Nemazee, A. B. Ward, I. A. Wilson, Structural and functional ramifications of antigenic drift in recent SARS-CoV-2 variants. *Science* **373**, 818–823 (2021). doi:10.1126/science.abh1139 Medline

67. K. R. McCarthy, L. J. Rennick, S. Nambulli, L. R. Robinson-McCarthy, W. G. Bain, G. Haidar, W. P. Duprex, Recurrent deletions in the SARS-CoV-2 spike glycoprotein drive antibody escape. *Science* **371**, 1139–1142 (2021). doi:10.1126/science.abf6950 Medline

68. B. Choi, M. C. Choudhary, J. Regan, J. A. Sparks, R. F. Padera, X. Qiu, I. H. Solomon, H. H. Kuo, J. Boucau, K. Bowman, U. D. Adhikari, M. L. Winkler, A. A. Mueller, T. Y. Hsu, M. Desjardins, L. R. Baden, B. T. Chan, B. D. Walker, M. Lichterfeld, M. Brigl, D. S. Kwon, S. Kanjilal, E. T. Richardson, A. H. Jonsson, G. Alter, A. K. Barczak, W. P. Hanage, X. G. Yu, G. D. Gaiha, M. S. Seaman, M. Cernadas, J. Z. Li, Persistence and

evolution of SARS-CoV-2 in an immunocompromised host. *N. Engl. J. Med.* **383**, 2291–2293 (2020). doi:10.1056/NEJMc2031364 Medline

69. V. A. Avanzato, M. J. Matson, S. N. Seifert, R. Pryce, B. N. Williamson, S. L. Anzick, K. Barbian, S. D. Judson, E. R. Fischer, C. Martens, T. A. Bowden, E. de Wit, F. X. Riedo, V. J. Munster, Case study: Prolonged infectious SARS-CoV-2 shedding from an asymptomatic immunocompromised individual with cancer. *Cell* **183**, 1901–1912.e9 (2020). doi:10.1016/j.cell.2020.10.049 Medline

70. A. Rosa, V. E. Pye, C. Graham, L. Muir, J. Seow, K. W. Ng, N. J. Cook, C. Rees-Spear, E. Parker, M. S. Dos Santos, C. Rosadas, A. Susana, H. Rhys, A. Nans, L. Masino, C. Roustan, E. Christodoulou, R. Ulferts, A. G. Wrobel, C.-E. Short, M. Fertleman, R. W. Sanders, J. Heaney, M. Spyer, S. Kjær, A. Riddell, M. H. Malim, R. Beale, J. I. MacRae, G. P. Taylor, E. Nastouli, M. J. van Gils, P. B. Rosenthal, M. Pizzato, M. O. McClure, R. S. Tedder, G. Kassiotis, L. E. McCoy, K. J. Doores, P. Cherepanov, SARS-CoV-2 can recruit a heme metabolite to evade antibody immunity. *Sci. Adv.* **7**, eabg7607 (2021). doi:10.1126/sciadv.abg7607 Medline

71. V.-V. Edara, B. A. Pinsky, M. S. Suthar, L. Lai, M. E. Davis-Gardner, K. Floyd, M. W. Flowers, J. Wrammert, L. Hussaini, C. R. Ciric, S. Bechnak, K. Stephens, B. S. Graham, E. Bayat Mokhtari, P. Mudvari, E. Boritz, A. Creanga, A. Pegu, A. Derrien-Colemyn, A. R. Henry, M. Gagne, D. C. Douek, M. K. Sahoo, M. Sibai, D. Solis, R. J. Webby, T. Jeevan, T. P. Fabrizio, Infection and vaccine-induced neutralizing-antibody responses to the SARS-CoV-2 B.1.617 variants. *N. Engl. J. Med.* **385**, 664–666 (2021). doi:10.1056/NEJMc2107799 Medline

72. D. Planas, D. Veyer, A. Baidaliuk, I. Staropoli, F. Guivel-Benhassine, M. M. Rajah, C. Planchais, F. Porrot, N. Robillard, J. Puech, M. Prot, F. Gallais, P. Gantner, A. Velay, J. Le Guen, N. Kassis-Chikhani, D. Edriss, L. Belec, A. Seve, L. Courtellemont, H. Péré, L. Hocqueloux, S. Fafi-Kremer, T. Prazuck, H. Mouquet, T. Bruel, E. Simon-Lorière, F. A. Rey, O. Schwartz, Reduced sensitivity of SARS-CoV-2 variant Delta to antibody neutralization. *Nature* **596**, 276–280 (2021). doi:10.1038/s41586-021-03777-9 Medline

73. A. Saito, T. Irie, R. Suzuki, T. Maemura, H. Nasser, K. Uriu, Y. Kosugi, K. Shirakawa, K. Sadamasu, I. Kimura, J. Ito, J. Wu, K. Iwatsuki-Horimoto, M. Ito, S. Yamayoshi, S. Ozono, E. P. Butlertanaka, Y. L. Tanaka, R. Shimizu, K. Shimizu, K. Yoshimatsu, R. Kawabata, T. Sakaguchi, K. Tokunaga, I. Yoshida, H. Asakura, M. Nagashima, Y. Kazuma, R. Nomura, Y. Horisawa, K. Yoshimura, A. Takaori-Kondo, M. Imai, S. Nakagawa, T. Ikeda, T. Fukuhara, Y. Kawaoka, K. Sato; The Genotype to Phenotype Japan (G2P-Japan) Consortium, SARS-CoV-2 spike P681R mutation, a hallmark of the Delta variant, enhances viral fusogenicity and pathogenicity. bioRxiv 2021.06.17.448820 [Preprint] (2021). https://doi.org/10.1101/2021.06.17.448820.

74. B. A. Johnson, X. Xie, A. L. Bailey, B. Kalveram, K. G. Lokugamage, A. Muruato, J. Zou, X. Zhang, T. Juelich, J. K. Smith, L. Zhang, N. Bopp, C. Schindewolf, M. Vu, A. Vanderheiden, E. S. Winkler, D. Swetnam, J. A. Plante, P. Aguilar, K. S. Plante, V. Popov, B. Lee, S. C. Weaver, M. S. Suthar, A. L. Routh, P. Ren, Z. Ku, Z. An, K. Debbink, M. S. Diamond, P.-Y. Shi, A. N. Freiberg, V. D. Menachery, Loss of furin cleavage site attenuates SARS-CoV-2 pathogenesis. *Nature* **591**, 293–299 (2021). doi:10.1038/s41586-021-03237-4 Medline

75. Y. J. Hou, K. Okuda, C. E. Edwards, D. R. Martinez, T. Asakura, K. H. Dinnon 3rd, T. Kato, R. E. Lee, B. L. Yount, T. M. Mascenik, G. Chen, K. N. Olivier, A. Ghio, L. V. Tse, S. R. Leist, L. E. Gralinski, A. Schäfer, H. Dang, R. Gilmore, S. Nakano, L. Sun, M. L. Fulcher, A. Livraghi-Butrico, N. I. Nicely, M. Cameron, C. Cameron, D. J. Kelvin, A. de Silva, D. M. Margolis, A. Markmann, L. Bartelt, R. Zumwalt, F. J. Martinez, S. P. Salvatore, A. Borczuk, P. R. Tata, V. Sontake, A. Kimple, I. Jaspers, W. K. O'Neal, S. H. Randell, R. C. Boucher, R. S. Baric, SARS-CoV-2 reverse genetics reveals a variable infection gradient in the respiratory tract. *Cell* **182**, 429–446.e14 (2020). doi:10.1016/j.cell.2020.05.042 Medline

76. C. Motozono, M. Toyoda, J. Zahradnik, A. Saito, H. Nasser, T. S. Tan, I. Ngare, I. Kimura, K. Uriu, Y. Kosugi, Y. Yue, R. Shimizu, J. Ito, S. Torii, A. Yonekawa, N. Shimono, Y. Nagasaki, R. Minami, T. Toya, N. Sekiya, T. Fukuhara, Y. Matsuura, G. Schreiber, T. Ikeda, S. Nakagawa, T. Ueno, K. Sato; Genotype to Phenotype Japan (G2P-Japan) Consortium, SARS-CoV-2 spike L452R variant evades cellular immunity and increases infectivity. *Cell Host Microbe* **29**, 1124–1136.e11 (2021). doi:10.1016/j.chom.2021.06.006 Medline

77. L. G. Thorne, M. Bouhaddou, A.-K. Reuschl, L. Zuliani-Alvarez, B. Polacco, A. Pelin, J. Batra, M. V. X. Whelan, M. Ummadi, A. Rojc, J. Turner, K. Obernier, H. Braberg, M. Soucheray, A. Richards, K.-H. Chen, B. Harjai, D. Memon, M. Hosmillo, J. Hiatt, A. Jahun, I. G. Goodfellow, J. M. Fabius, K. Shokat, N. Jura, K. Verba, M. Noursadeghi, P. Beltrao, D. L. Swaney, A. Garcia-Sastre, C. Jolly, G. J. Towers, N. J. Krogan, Evolution of enhanced innate immune evasion by the SARS-CoV-2 B.1.1.7 UK variant. bioRxiv 2021.06.06.446826 [Preprint] (2021). https://doi.org/10.1101/2021.06.06.446826.

78. A. Z. Wec, D. Wrapp, A. S. Herbert, D. P. Maurer, D. Haslwanter, M. Sakharkar, R. K. Jangra, M. E. Dieterle, A. Lilov, D. Huang, L. V. Tse, N. V. Johnson, C. L. Hsieh, N. Wang, J. H. Nett, E. Champney, I. Burnina, M. Brown, S. Lin, M. Sinclair, C. Johnson, S. Pudi, R. Bortz 3rd, A. S. Wirchnianski, E. Laudermilch, C. Florez, J. M. Fels, C. M. O'Brien, B. S. Graham, D. Nemazee, D. R. Burton, R. S. Baric, J. E. Voss, K. Chandran, J. M. Dye, J. S. McLellan, L. M. Walker, Broad neutralization of SARS-related viruses by human monoclonal antibodies. *Science* **369**, 731–736 (2020). doi:10.1126/science.abc7424 Medline

79. C. G. Rappazzo, L. V. Tse, C. I. Kaku, D. Wrapp, M. Sakharkar, D. Huang, L. M. Deveau, T. J. Yockachonis, A. S. Herbert, M. B. Battles, C. M. O'Brien, M. E. Brown, J. C. Geoghegan, J. Belk, L. Peng, L. Yang, Y. Hou, T. D. Scobey, D. R. Burton, D. Nemazee, J. M. Dye, J. E. Voss, B. M. Gunn, J. S. McLellan, R. S. Baric, L. E. Gralinski, L. M. Walker, Broad and potent activity against SARS-like viruses by an engineered human monoclonal antibody. *Science* **371**, 823–829 (2021). doi:10.1126/science.abf4830 Medline

80. D. R. Martinez, A. Schaefer, S. Gobeil, D. Li, G. De la Cruz, R. Parks, X. Lu, M. Barr, K. Manne, K. Mansouri, R. J. Edwards, B. Yount, K. Anasti, S. A. Montgomery, S. Shen, T. Zhou, P. D. Kwong, B. S. Graham, J. R. Mascola, D. C. Montefiori, M. Alam, G. D. Sempowski, K. Wiehe, K. O. Saunders, P. Acharya, B. F. Haynes, R. S. Baric, A broadly neutralizing antibody protects against SARS-CoV, pre-emergent bat CoVs, and SARS-

CoV-2 variants in mice. bioRxiv 2021.04.27.441655 [Preprint] (2021). https://doi.org/10.1101/2021.04.27.441655.

81. D. R. Martinez, A. Schäfer, S. R. Leist, G. De la Cruz, A. West, E. N. Atochina-Vasserman, L. C. Lindesmith, N. Pardi, R. Parks, M. Barr, D. Li, B. Yount, K. O. Saunders, D. Weissman, B. F. Haynes, S. A. Montgomery, R. S. Baric, Chimeric spike mRNA vaccines protect against Sarbecovirus challenge in mice. *Science* **373**, 991–998 (2021). doi:10.1126/science.abi4506 Medline

82. A. A. Cohen, P. N. P. Gnanapragasam, Y. E. Lee, P. R. Hoffman, S. Ou, L. M. Kakutani, J. R. Keeffe, H.-J. Wu, M. Howarth, A. P. West, C. O. Barnes, M. C. Nussenzweig, P. J. Bjorkman, Mosaic nanoparticles elicit cross-reactive immune responses to zoonotic coronaviruses in mice. *Science* **371**, 735–741 (2021). doi:10.1126/science.abf6840 Medline

83. A. C. Walls, B. Fiala, A. Schäfer, S. Wrenn, M. N. Pham, M. Murphy, L. V. Tse, L. Shehata, M. A. O'Connor, C. Chen, M. J. Navarro, M. C. Miranda, D. Pettie, R. Ravichandran, J. C. Kraft, C. Ogohara, A. Palser, S. Chalk, E. C. Lee, K. Guerriero, E. Kepl, C. M. Chow, C. Sydeman, E. A. Hodge, B. Brown, J. T. Fuller, K. H. Dinnon 3rd, L. E. Gralinski, S. R. Leist, K. L. Gully, T. B. Lewis, M. Guttman, H. Y. Chu, K. K. Lee, D. H. Fuller, R. S. Baric, P. Kellam, L. Carter, M. Pepper, T. P. Sheahan, D. Veesler, N. P. King, Elicitation of potent neutralizing antibody responses by designed protein nanoparticle vaccines for SARS-CoV-2. *Cell* **183**, 1367–1382.e17 (2020). doi:10.1016/j.cell.2020.10.043 Medline

84. C. J. Russo, L. A. Passmore, Ultrastable gold substrates for electron cryomicroscopy. *Science* **346**, 1377–1380 (2014). doi:10.1126/science.1259530 Medline

85. C. Suloway, J. Pulokas, D. Fellmann, A. Cheng, F. Guerra, J. Quispe, S. Stagg, C. S. Potter, B. Carragher, Automated molecular microscopy: The new Leginon system. *J. Struct. Biol.* **151**, 41–60 (2005). doi:10.1016/j.jsb.2005.03.010 Medline

86. D. Tegunov, P. Cramer, Real-time cryo-electron microscopy data preprocessing with Warp. *Nat. Methods* **16**, 1146–1152 (2019). doi:10.1038/s41592-019-0580-y Medline

87. A. Punjani, J. L. Rubinstein, D. J. Fleet, M. A. Brubaker, cryoSPARC: Algorithms for rapid unsupervised cryo-EM structure determination. *Nat. Methods* **14**, 290–296 (2017). doi:10.1038/nmeth.4169 Medline

88. A. Punjani, H. Zhang, D. J. Fleet, Non-uniform refinement: Adaptive regularization improves single-particle cryo-EM reconstruction. *Nat. Methods* **17**, 1214–1221 (2020). doi:10.1038/s41592-020-00990-8 Medline

89. J. Zivanov, T. Nakane, S. H. W. Scheres, A Bayesian approach to beam-induced motion correction in cryo-EM single-particle analysis. *IUCrJ* **6**, 5–17 (2019). doi:10.1107/S205225251801463X Medline

90. J. Zivanov, T. Nakane, B. O. Forsberg, D. Kimanius, W. J. Hagen, E. Lindahl, S. H. Scheres, New tools for automated high-resolution cryo-EM structure determination in RELION-3. *eLife* **7**, e42166 (2018). doi:10.7554/eLife.42166 Medline

91. S. H. Scheres, RELION: Implementation of a Bayesian approach to cryo-EM structure determination. *J. Struct. Biol.* **180**, 519–530 (2012). doi:10.1016/j.jsb.2012.09.006 Medline

92. P. B. Rosenthal, R. Henderson, Optimal determination of particle orientation, absolute hand, and contrast loss in single-particle electron cryomicroscopy. *J. Mol. Biol.* **333**, 721–745 (2003). doi:10.1016/j.jmb.2003.07.013 Medline

93. S. Chen, G. McMullan, A. R. Faruqi, G. N. Murshudov, J. M. Short, S. H. Scheres, R. Henderson, High-resolution noise substitution to measure overfitting and validate resolution in 3D structure determination by single particle electron cryomicroscopy. *Ultramicroscopy* **135**, 24–35 (2013). doi:10.1016/j.ultramic.2013.06.004 Medline

94. E. F. Pettersen, T. D. Goddard, C. C. Huang, G. S. Couch, D. M. Greenblatt, E. C. Meng, T. E. Ferrin, UCSF Chimera—A visualization system for exploratory research and analysis. *J. Comput. Chem.* **25**, 1605–1612 (2004). doi:10.1002/jcc.20084 Medline

95. P. Emsley, B. Lohkamp, W. G. Scott, K. Cowtan, Features and development of *Coot*. *Acta Crystallogr. D* **66**, 486–501 (2010). doi:10.1107/S0907444910007493 Medline

96. B. Frenz, S. Rämisch, A. J. Borst, A. C. Walls, J. Adolf-Bryfogle, W. R. Schief, D. Veesler, F. DiMaio, Automatically fixing errors in glycoprotein structures with Rosetta. *Structure* **27**, 134–139.e3 (2019). doi:10.1016/j.str.2018.09.006 Medline

97. R. Y. Wang, Y. Song, B. A. Barad, Y. Cheng, J. S. Fraser, F. DiMaio, Automated structure refinement of macromolecular assemblies from cryo-EM maps using Rosetta. *eLife* **5**, e17219 (2016). doi:10.7554/eLife.17219 Medline

98. D. Liebschner, P. V. Afonine, M. L. Baker, G. Bunkóczi, V. B. Chen, T. I. Croll, B. Hintze, L. W. Hung, S. Jain, A. J. McCoy, N. W. Moriarty, R. D. Oeffner, B. K. Poon, M. G. Prisant, R. J. Read, J. S. Richardson, D. C. Richardson, M. D. Sammito, O. V. Sobolev, D. H. Stockwell, T. C. Terwilliger, A. G. Urzhumtsev, L. L. Videau, C. J. Williams, P. D. Adams, Macromolecular structure determination using X-rays, neutrons and electrons: Recent developments in *Phenix*. *Acta Crystallogr. D* **75**, 861–877 (2019). doi:10.1107/S2059798319011471 Medline

99. T. I. Croll, *ISOLDE*: A physically realistic environment for model building into low-resolution electron-density maps. *Acta Crystallogr. D* **74**, 519–530 (2018). doi:10.1107/S2059798318002425 Medline

100. V. B. Chen, W. B. Arendall 3rd, J. J. Headd, D. A. Keedy, R. M. Immormino, G. J. Kapral, L. W. Murray, J. S. Richardson, D. C. Richardson, *MolProbity*: All-atom structure validation for macromolecular crystallography. *Acta Crystallogr. D* **66**, 12–21 (2010). doi:10.1107/S0907444909042073 Medline

101. B. A. Barad, N. Echols, R. Y. Wang, Y. Cheng, F. DiMaio, P. D. Adams, J. S. Fraser, EMRinger: Side chain-directed model and map validation for 3D cryo-electron microscopy. *Nat. Methods* **12**, 943–946 (2015). doi:10.1038/nmeth.3541 Medline

102. J. Agirre, J. Iglesias-Fernández, C. Rovira, G. J. Davies, K. S. Wilson, K. D. Cowtan, Privateer: Software for the conformational validation of carbohydrate structures. *Nat. Struct. Mol. Biol.* **22**, 833–834 (2015). doi:10.1038/nsmb.3115 Medline

103. T. D. Goddard, C. C. Huang, E. C. Meng, E. F. Pettersen, G. S. Couch, J. H. Morris, T. E. Ferrin, UCSF ChimeraX: Meeting modern challenges in visualization and analysis. *Protein Sci.* **27**, 14–25 (2018). doi:10.1002/pro.3235 Medline
